# Deletion of DDB1- and CUL4- associated factor-17 (*Dcaf17)* gene causes spermatogenesis defects and male infertility in mice

**DOI:** 10.1038/s41598-018-27379-0

**Published:** 2018-06-15

**Authors:** Asmaa Ali, Bhavesh V. Mistry, Hala A. Ahmed, Razan Abdulla, Hassan A. Amer, Abdelbary Prince, Anas M. Alazami, Fowzan S. Alkuraya, Abdullah Assiri

**Affiliations:** 1 Comparative Medicine Department, King Faisal Specialist Hospital and Research Centre, Riyadh, 11211 Saudi Arabia; 2 Department of Genetics, King Faisal Specialist Hospital and Research Centre, Riyadh, 11211 Saudi Arabia; 3 Department of Biochemistry and Molecular Biology, Faculty of Veterinary Medicine, Cairo University, Giza, 12613 Egypt; 4 College of Medicine, AlFaisal University, Riyadh, Saudi Arabia; 5 Institute for Research and Medical Consultations, Imam Abdulrahman Bin Faisal University, Dammam, Saudi Arabia

## Abstract

DDB1– and CUL4–associated factor 17 (*Dcaf17*) is a member of DCAF family genes that encode substrate receptor proteins for Cullin-RING E3 ubiquitin ligases, which play critical roles in many cellular processes. To unravel the function of DCAF17, we performed expression profiling of *Dcaf17* in different tissues of wild type mouse by qRT-PCR and generated *Dcaf17* knockout mice by gene targeting. Expression profiling of *Dcaf17* showed highest expression in testis. Analyses of *Dcaf17* transcripts during post-natal development of testis at different ages displayed gradual increase in *Dcaf17* mRNA levels with the age. Although *Dcaf17* disruption did not have any effect on female fertility, *Dcaf17* deletion led to male infertility due to abnormal sperm development. The *Dcaf17*^*−/−*^ mice produced low number of sperm with abnormal shape and significantly low motility. Histological examination of the *Dcaf17*^*−/−*^ testis revealed impaired spermatogenesis with presence of vacuoles and sloughed cells in the seminiferous tubules. Disruption of *Dcaf17* caused asymmetric acrosome capping, impaired nuclear compaction and abnormal round spermatid to elongated spermatid transition. For the first time, these data indicate that DCAF17 is essential for spermiogenesis.

## Introduction

Infertility is a global health problem that affects 15% of couples worldwide^1,2^. Almost half of these infertility cases are attributed to male infertility, where about 75% of all the male factor infertility attributed to defective spermatogenesis^3–5^. Despite of advancement in the fields of biomedicine and genetics, the underlying causes of male infertility remain largely unknown in about 50% cases and they are categorized as idiopathic infertility cases^1^.

Mammalian spermatogenesis is a complex and highly regulated process that occurs within the seminiferous tubules, where the pluripotent spermatogonial stem cells proliferate through mitotic cell divisions and differentiate into primary spermatocytes. Primary spermatocytes then undergo two meiotic divisions to produce haploid round spermatids, which subsequently differentiate into highly specialized spermatozoa by a unique metamorphosis process called spermiogenesis^6,7^. During spermiogenesis, many biochemical and structural changes necessary for the formation of acrosome, nucleus elongation, chromatin condensation, formation of flagellum and removal of cytoplasmic residual body are carried out in an intricate manner^8–10^. Normal spermatogenesis requires regulated protein homeostasis and disruption of such balance results in male infertility^11–13^.

The ubiquitin-proteasome system (UPS) plays an important role in numerous developmental processes, including spermatogenesis, by selective and timely protein turnover for proper cellular functions in eukaryotes^12,14–16^. In UPS, ubiquitin is covalently transferred to one or more lysine residues of the target protein through a series of enzymatic reactions involving ubiquitin-activating enzyme (E1), ubiquitin-conjugating enzyme (E2) and ubiquitin ligase enzyme (E3)^15,17,18^. E3 ubiquitin ligases (E3s) are diverse enzymes with modular, single protein or multi-protein complex structures that provide specificity toward a variety of substrates for the ubiquitination^19–21^. Cullin (CUL) 4 A and CUL4B, members of cullin-RING finger E3 ligase (CRL) sub-family, have been shown to play distinct and essential roles during spermatogenesis^22–25^. Both the proteins have displayed complementary expression patterns in adult mouse testis, where CUL4A is mainly present in meiosis-stage spermatocytes, while CUL4B is predominantly expressed in Sertoli cells, spermatogonia and spermatids^24^. Disruption of *Cul4A* caused male infertility because of the defective meiotic progression^24,25^. Whereas, the male infertility phenotype resulted from *Cul4B* deletion was due to abnormal post-meiotic sperm development^22,23^.

CUL4 proteins interact with the E2 enzyme via the RING finger protein Hrt1/ROC1/Rbx1 at their C terminus. Whereas at their N terminus, they employ DDB1 (DNA damage-binding protein 1), as an adaptor protein, which in turn recruits different substrate receptor proteins known as DDB1- and CUL4-associated factors (DCAFs)^26–28^. These DCAFs have been suggested to determine the substrate specificity in different CUL4-DDB1-based E3 ligase complexes^27^. Mutations in *DCAF17* gene caused an extremely rare autosomal recessive disorder known as Woodhouse-Sakati syndrome (WSS) in human^29^. Patients with WSS display hypogonadism, alopecia, diabetes, mental retardation, deafness and extrapyramidal symptoms^29–31^. Male patients with *DCAF17* mutations showed azoospermia with very less germ cell mass and large number of Sertoli cells in testicular biopsies^30,31^. However, the role of DCAF17 in the male reproductive system is not clear. To investigate the role of DCAF17 protein in mammalian spermatogenesis, we generated and characterized global *Dcaf17* mutant mice. Our data show that *Dcaf17* mutant male mice are infertile and the DCAF17 protein is required for normal spermiogenesis process and sperm functionality.

## Results

### Levels of *Dcaf17* mRNA in different tissues of mice

**Figure 1:**
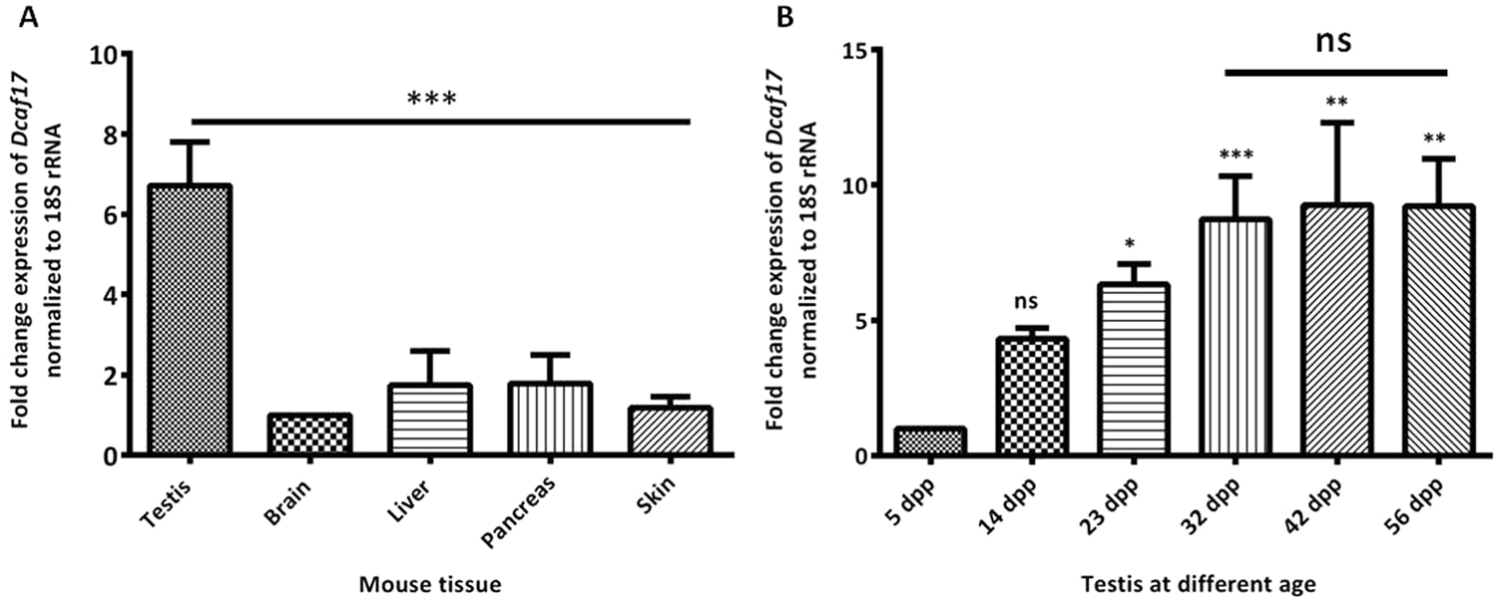
Investigating *Dcaf17* mRNA levels by real time PCR. (**A**) Relative expression of *Dcaf17* in different tissues of adult mouse. Relative expression values of *Dcaf17* mRNA were normalized to 18S rRNA expression and the level of *Dcaf17* mRNA in the brain was arbitrarily set at 1. The *Dcaf17* is highly expressed in testis, while other tissues show low level of expression. (**B**) Relative expression of *Dcaf17* in testis at different ages. Relative expression values of *Dcaf17* mRNA were normalized to 18S rRNA expression and the level of *Dcaf17* mRNA in the 5 days postpartum (dpp) testis was arbitrarily set at 1. Expression of *Dcaf17* in the testis increased with the age till the age of 32 dpp and then after it remained constant. Asterisks indicate statistical significance (p < 0.0001 for A and p < 0.0036 for B). Error bars represent SEM. Three to five animals were used to investigate the transcript levels of *Dcaf17*. Experiments were performed in triplicates. Data were analyzed on Graphpad prism 5 software using one-way ANOVA technique followed by post hoc Bonferroni’s (**A**) or Tukey’s (**B**) multiple comparison test. ns - not significant.

The *Dcaf17* mRNA transcript profile was examined by relative qRT-PCR analysis in five different tissues that included brain, liver, pancreas, skin and testis from adult mouse. The level of *Dcaf17* transcripts in brain was arbitrarily chosen as a calibrator and standardized to one. The 18S rRNA expression was used as endogenous control. The fold change in the *Dcaf17* mRNA levels, normalized to 18S rRNA and relative to brain, is reported (Fig. 1A). Levels of the *Dcaf17* mRNA transcript varied among the tissue samples tested. Testis showed highest level of the *Dcaf17* mRNA with almost 7 fold higher levels relative to that of the brain (Fig. 1A). Pancreas and liver showed only 1.7 fold higher mRNA levels of *Dcaf17* relative to that of the brain. However, the abundance of *Dcaf17* mRNA in skin was similar to that in the brain (Fig. 1A).

Further to examine the *Dcaf17* mRNA transcript levels in testis at different postnatal developmental stages, relative qRT-PCR was performed on the testes total RNA samples collected at 5, 14, 23, 32, 42 and 56 days postpartum (dpp). The *Dcaf17* mRNA level at Day 5 testis was chosen as a calibrator and standardized to one. The 18S rRNA levels were used as endogenous control. The fold change in the *Dcaf17* gene expression, normalized to 18S rRNA and relative to 5 dpp testis, is reported (Fig. 1B). The *Dcaf17* transcript abundance in testis gradually increased with the age until 32 dpp and plateau thereafter (Fig. 1B).

### Generation of *Dcaf17* KO mice

**Figure 2:**
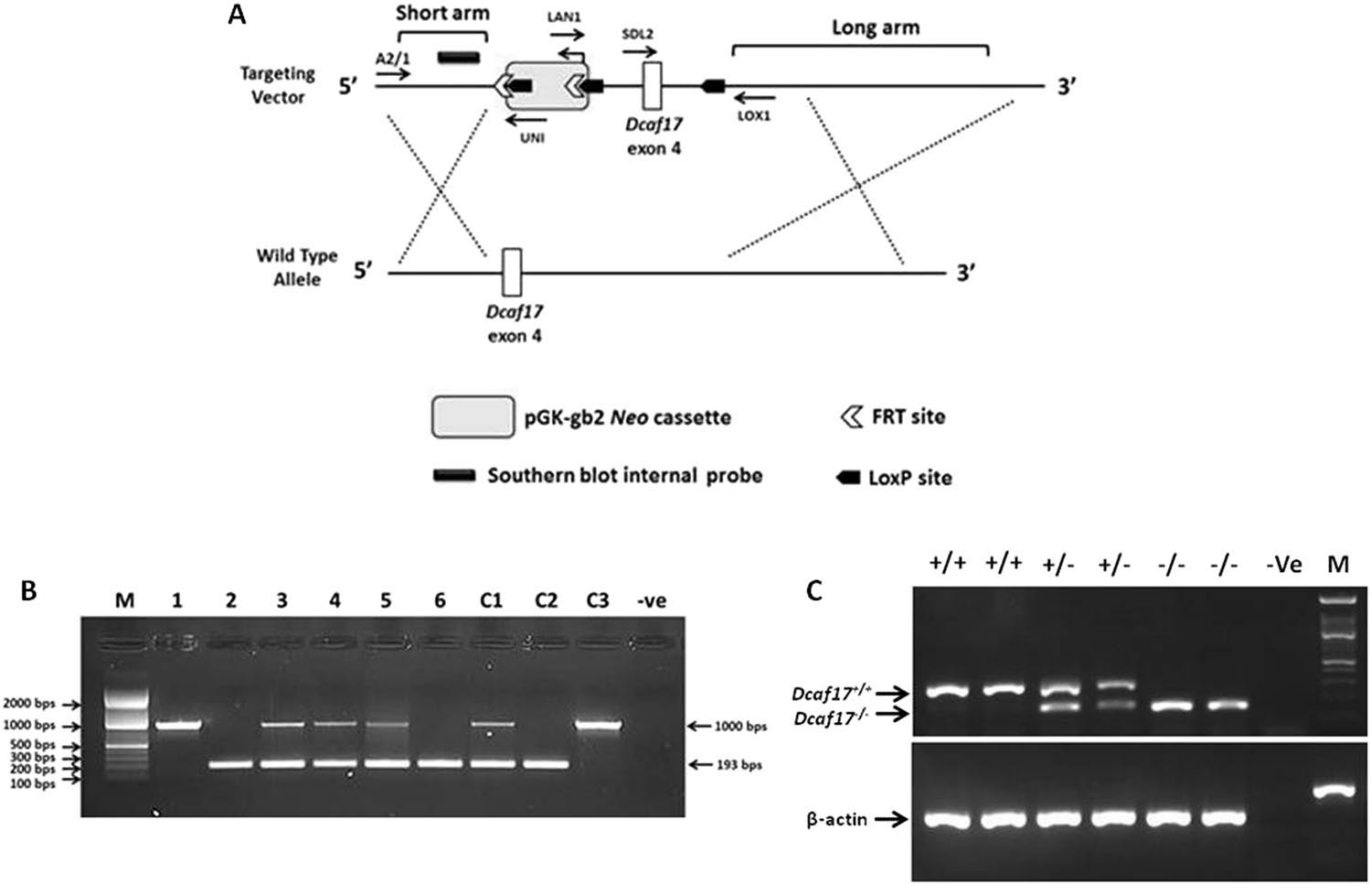
Diagrammatic representation (A) of *Dcaf17* gene targeting approach in mouse by homologous recombination and genotyping (**B**,**C**) of different alleles of *Dcaf17* in mice. (**A**) Homologous recombination strategy in mouse ES cells. The *Dcaf17* targeting vector (top) was constructed to replace wild type exon 4 and introduce neomycin drug selection marker, *LoxP* and FRT sites. (**B**) Agarose gel image of PCR genotyping of representative *Dcaf17* mutant mice. PCR amplification of wild type genotype gives 1 kbps amplicon (1, C3), heterozygous genotype for *Dcaf17* mutation gives 1 kbps and 193 bps amplicons (3–5, C1) and homozygous genotype for *Dcaf17* mutation gives 193 bps amplicon (2, 6 and C2). 1–6 – genomic DNA samples of different *Dcaf17* genotypes; C1-C3 – Different *Dcaf17* genotype controls; −Ve – no template control. (**C**) Agarose gel image of RT-PCR of different *Dcaf17* genotypes. PCR products of various *Dcaf17* alleles and β-actin were run on the same agarose gel and single image was taken. +/+ - *Dcaf17*^+/+^ (WT); +/− - *Dcaf17*^+/−^ (heterozygous *Dcaf17* mutant); −/− - *Dcaf17*^*−/−*^ (homozygous *Dcaf17* mutant); −Ve – no template control; M – DNA ladder. PCR fragment size for β-actin is 190 bps; for *Dcaf17*^+/+^ is 284 bps and for *Dcaf17*^*−/−*^ is 148 bps. Gel images were taken using ImageQuant LAS 4000 imaging system.

To investigate the role of the *Dcaf17* gene *in vivo*, knockout (KO) mice were created by deleting exon 4 of the *Dcaf17* using gene targeting approach (Fig. S1; Fig. S2A,B). The *Dcaf17*^*fl/fl*^ mice were confirmed by PCR and Southern blotting (Fig. S2D–F). The *Dcaf17*^*−/−*^ mice were generated by crossing *Dcaf17*^*fl/fl*^ mice with CMV-*Cre* transgenic mice to delete exon 4 of *Dcaf17* gene. It was found that the mutant allele for *Dcaf17* gene could be transmitted from both *Dcaf17* heterozygous males and females, and the homozygous mice could be obtained from interbreeding of the heterozygous male and female mice (Fig. 2B,C). Deletion of exon 4 of *Dcaf17* gene was confirmed by PCR, RT-PCR (Fig. 2B,C) and cDNA sequencing analyses (Fig. S3). The cDNA sequencing analysis of *Dcaf17*^*−/−*^ mice revealed that excision of exon 4 led to a frameshift mutation (NM_001165980.1:c.322_458del:p.G108Vfs*59) with concomitant premature stop codon after 59 residues (Fig. S3). All the conditional, heterozygous and homozygous *Dcaf17* KO mice (male and female) were viable and showed no gross abnormalities. Due to male infertility, the colony was maintained through heterozygous mating system and continuous pups’ genotyping.

### Male *Dcaf17*^*−/−*^ KO mice are infertile

To assess the fertility phenotype of *Dcaf17* KO mice, controlled breeding experiments were carried out where adult WT and *Dcaf17*^*−/−*^ males were individually mated with WT or *Dcaf17*^*−/−*^ adult females. Mating behavior of WT and *Dcaf17*^*−/−*^ was monitored by checking the ability to produce vaginal (copulatory) plugs in the female mice. All the females with WT or *Dcaf17*^*−/−*^ genotype produced vaginal plugs after mating either with WT or *Dcaf17*^*−/−*^ male mice, suggesting normal mating behavior among both the genotypes (Table 1).

**Table 1:** Fertility testing of *Dcaf17*^−/−^ and wild type mice.

Genotype		Vaginal plug	Total No. of litters	Total No. of pups	Average litter size ± SD
Male (n)	Females (n)				
*Dcaf17* ^+/+^ *(3)*	*Dcaf17* ^+/+^ *(3)*	+	13	46	3.54 ± 1.6
*Dcaf17* ^*−/−*^ *(5)*	*Dcaf17* ^+/+^ *(5)*	+	0	0	0
*Dcaf17* ^+/+^ *(3)*	*Dcaf17* ^−/−^ *(3)*	+	8	25	3.13 ± 1.6
*Dcaf17* ^*−/−*^ *(3)*	*Dcaf17* ^−/−^ *(3)*	+	0	0	0

n - Number of animals used; + - Vaginal plug positive; SD - Standard Deviation.

Breeding three WT females and three WT males produced 46 pups in 13 litters with an average litter size of 3.54 pups (Table 1). Whereas, five WT females plugged by five *Dcaf17*^*−/−*^ males neither displayed any evidence of pregnancy nor produced any pups within the observation period (Table 1). Similarly, three *Dcaf17* homozygous mutant female mated with three individual *Dcaf17*^*−/−*^ male did not become pregnant and produce pups. Unlike male *Dcaf17*^*−/−*^ mice, the three *Dcaf17*^*−/−*^ female mice plugged by three WT males produced 25 pups in 8 litters with an average of 3.13 pups per litter (Table 1). Statistical analysis of the litter size of WT male and WT female mice breeding with that of WT male and *Dcaf17* KO female breeding showed no significant difference between the litter size (P value ≤ 0.15), suggesting that there is no effect of *Dcaf17* deletion on female fertility in the mice. All the *Dcaf17*^+/−^ heterozygous KO mice were fertile and produced comparable number of pups with that of WT mice (Data not shown). These data show that *Dcaf17* homozygous KO male mice are infertile and the infertility phenotype did not result from abnormal sexual behavior but resulted from defective testicular function.

### *Dcaf17*^*−/−*^ mice have severely reduced sperm production and motility

**Figure 3:**
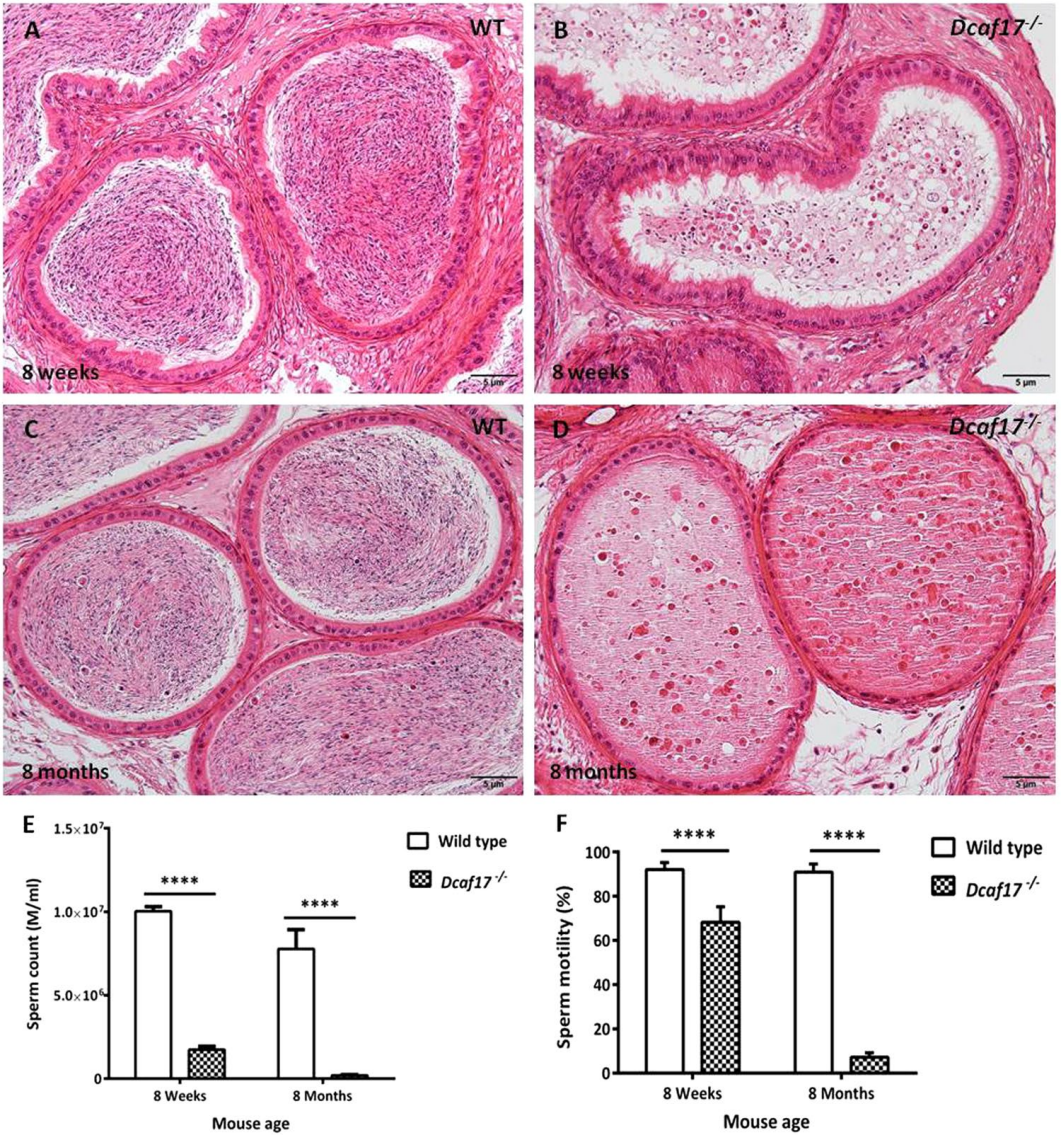
Histology of cauda epididymides and cauda sperm analyses from WT and *Dcaf17*^*−/−*^ adult mice. Hematoxylin and eosin (**H**,**E**) staining of cauda epididymides sections (5 µm thick) from 8 weeks (**A**,**B**) and 8 months (**C**,**D**) old WT (**A**,**C**) and *Dcaf17*^*−/−*^ (**B**,**D**) mice. The lumen of WT cauda epididymis (**A**,**C**) is full of mature sperm, whereas the lumen of *Dcaf17*^*−/−*^ mutant cauda epididymidis (**B**,**D**) contains oval shaped sloughed cells of various size. Sperm count (**E**) and motility (**F**) analyses of WT and *Dcaf17*^*−/−*^ mutant mice at the age of 8 weeks and 8 months old reveal severe and progressive reduction in sperm count (**E**) and motility (**F**) in *Dcaf17*^*−/−*^ mice compared to WT mice. Around 300 sperm in a total of five fields in each replicate were analyze for sperm motility analysis. Number of animals used for each genotype to analyze sperm count (**E**) and motility (**F**) was 5. Data were analyzed on Graphpad prism 5 using two-way ANOVA technique followed by post hoc Bonferroni’s multiple comparison test. For images A to D the magnification is 200X and the scale bars are 5 µm. Error bars in panels E and F represent SEM. Asterisks in E and F indicate statistical significance (p < 0.0001 for E and F).

Breeding experiments of *Dcaf17* homozygous KO mice revealed that *Dcaf17*^*−/−*^ male mice are infertile. Morphological examination of the wild type and *Dcaf17* mutant testes showed no gross differences in the shape, size and weight of the testes from both the genotypes (Fig. S4; Table S1). Further to investigate how *Dcaf17* deficiency leads to male infertility, we first examined the cauda epididymal sperm of WT and *Dcaf17*^*−/−*^ adult mice. Histological examination of the WT and *Dcaf17*^*−/−*^ adult mice cauda epididymides revealed severe reduction in sperm amount within the lumen of *Dcaf17*^*−/−*^ cauda epididymis as compared to that of WT adult mice (Fig. 3A–D). Unlike WT cauda epididymis, where the lumen was full of normal mature sperm (Fig. 3A,C), the lumen of *Dcaf17*^*−/−*^ cauda epididymis showed very few spermatozoa, most of which were morphologically abnormal, and an unusual occurrence of abnormal cellular debris including numerous deeply stained round structures of various sizes, most likely germ cells that had sloughed off prematurely from the seminiferous tubules (Fig. 3B,D). Moreover, the lumen of cauda epididymis in 8 months old *Dcaf17* KO mouse was filled with abnormal cellular debris including numerous deeply stained round structures of various sizes, most likely degrading germ cells (Fig. 3D). No obvious differences in the structure of the epididymal epithelium were apparent between *Dcaf17* KO and control mice. Sperm count and motility analyses revealed significant reduction in the number and motility of *Dcaf17*^*−/−*^ epididymal sperm in comparison to that of WT mice (Fig. 3E,F). Eight weeks old *Dcaf17* KO mice produced almost six fold less sperm (1.7 ± 0.46 × 10^6^) with 68% motility as compared to the WT mice of the same age that produced 10 ± 6.3 × 10^6^ sperm with more than 90% motility (Fig. 3E,F). More reductions in the epididymal sperm count and percentage motility were observed in *Dcaf17*^*−/−*^ mice with advancing age. Eight months old *Dcaf17* KO mice produced almost 8.5 fold less sperm (0.20 ± 0.1 × 10^6^) compared to that of 8 weeks *Dcaf17* KO mice and 35 fold less sperm than their age-matched WT control. Eight months old WT showed about 1.4 fold sperm count reduction (7 ± 2.57 × 10^6^) in comparison to their genotype counterpart at 8 weeks of age (Fig. 3E,F).

### *Dcaf17*^*−/−*^ sperm defects are similar to those found in oligoasthenoteratozoospermia

**Figure 4:**
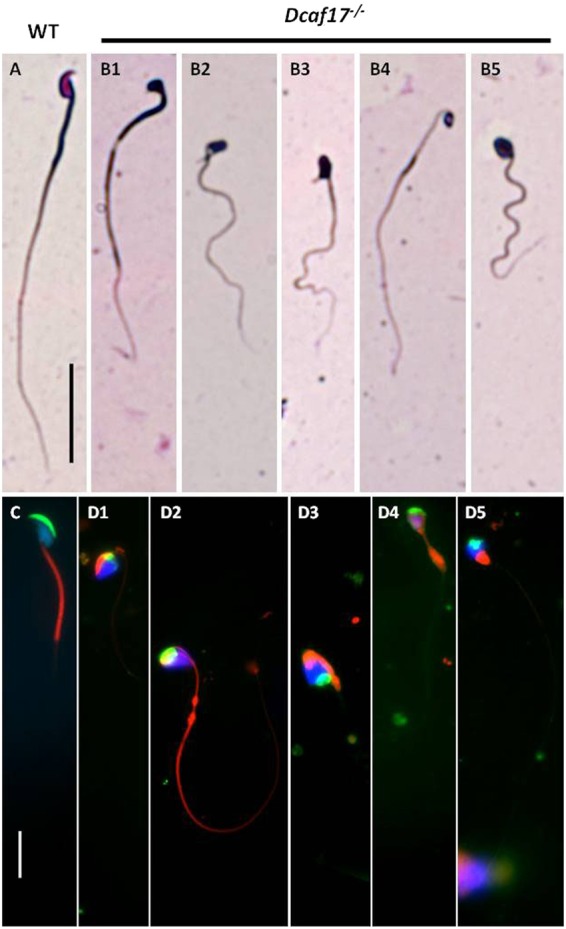
Bright field and fluorescence microscopy of cauda epididymal sperm from WT and *Dcaf17*^*−/−*^ adult mice. Representative images of Diff-Quick staining (bright field images, top panels) and MitoTracker staining (fluorescence images, bottom panels) of epididymal sperm spreads from WT (**A**,**C**) and *Dcaf17*^*−/−*^ (**B**,**D**) adult mice. The WT sperm (**A**,**C**) show typical hook-shaped head, patent midpiece and tail morphology. Whereas, the Dcaf17^−/−^ sperm (**B**,**D**) show variety of morphological defects in head shape, midpiece and tail. To categorize different sperm defects in *Dcaf17* KO mice we analyzed total 150 sperms from 3 different mice. Major categories of sperm head defects were triangular (cupcake) (**B1**,**D1**,**D3**), oval (**B5**,**D2**,**D4**,**D5**) or amorphous (**B2**,**B3**) shaped with many times either bent head or coiled midpiece surrounding the head. Fluorescently labeled WT sperm (**C**) shows normal crescent shaped acrosome (green), uniformly distributed mitochondria (red) along the midpiece and highly condensed nucleus (blue). *Dcaf17*^*−/−*^ sperm (**D1–5**) show spatially dysmorphic sperm structure with abnormal acrosome (green), ectopic localization of mitochondria (red) and diffused chromatin structure (blue). Images C and D1–5 are merged fluorescence images of sperm stained for acrosome (green), midpiece (red) and nucleus (blue). Separate fluorescence images for each sperm acrosome, midpiece and nucleus staining are shown in supplementary figure S5. Magnification of bright images (**A**,**B1–5**) is 400X and magnification of fluorescence images (**C**,**D1–5**) is 1000X. Scale bar in the image A is 1 mm and in the image C is 10 µm.

To examine *Dcaf17*^*−/−*^ mutant sperm morphology, sperm smears were prepared and stained with Diff-Quick stain. Sperms from WT male mice showed normal morphology, with characteristic hook-shaped head, midpiece and tail (Fig. 4A). Whereas, all the sperm of *Dcaf17*^*−/−*^ mice showed variety of defective morphology with abnormally shaped head containing deformed nucleus (Fig. 4B1–5). Further to analyze *Dcaf17*^*−/−*^ sperm defects in more detail, WT and mutant sperm were stained with Mito tracker (red), which labels mitochondria; FITC-labeled lectin PNA (green), which labels acrosome; and DAPI (blue), which labels the nucleus (Fig. 4C and D1–5). As shown in the Fig. 4C, WT sperm displayed hook-shaped head with typical crescent-like acrosome (green) covering the anterior surface of the nucleus (blue), and a mid-piece that is covered by mitochondrial sheath (red). In contrast to WT sperm, the acrosome of *Dcaf17*^*−/−*^ sperm failed to acquire the typical crescentic shape and exhibited various defects, including miss-localization and deformation (Figs. 4D1–5). The nucleus (blue) of WT mature sperm was highly condensed occupying most of the head space (Fig. 4C). In the *Dcaf17*^*−/−*^ mutant sperm, deformed and less condensed nucleus (blue) was observed (Fig. 4D1–5). The mitochondrial sheath (red), which is responsible for sperm movement, also exhibited various defects in *Dcaf17*^*−/−*^ mutant sperm, including mitochondria aggregating near the deformed nucleus, splitting into two separate aggregates and in many cases mid piece coiling around the deformed head (Fig. 4D1–5). We divided the observable sperm head shape defects into three major categories (Fig. 4B1–5, D1–5) and quantified their incidence by observing total number of 150 sperm randomly from *Dcaf17* KO mice. Our observation revealed that around 45% sperm had triangular (cupcake) shaped head (Fig. 4B1,D1,D3), 27% of the sperms had oval shaped head (Fig. 4B5,D2,D4,D5) and 28% of the sperms had amorphous head shape (Fig. 4B2,B3). Among all the sperm in the above described head shape defect categories, 80% of the sperm head contained acrosome which was miss-localized and abnormally formed. Acrosome was missing in the remaining 20% of sperm in all three categories analyzed. Tail analysis of the *Dcaf17*^*−/−*^ sperm in all three categories showed that in 38% sperm, the midpiece was coiled around sperm head, in 27% sperm had bent head from neck and remaining 35% sperm had normal straight head. Sperm with acrosome, nucleus and midpiece combinatory defects were not observed in WT or heterozygous mice.

**Figure 5:**
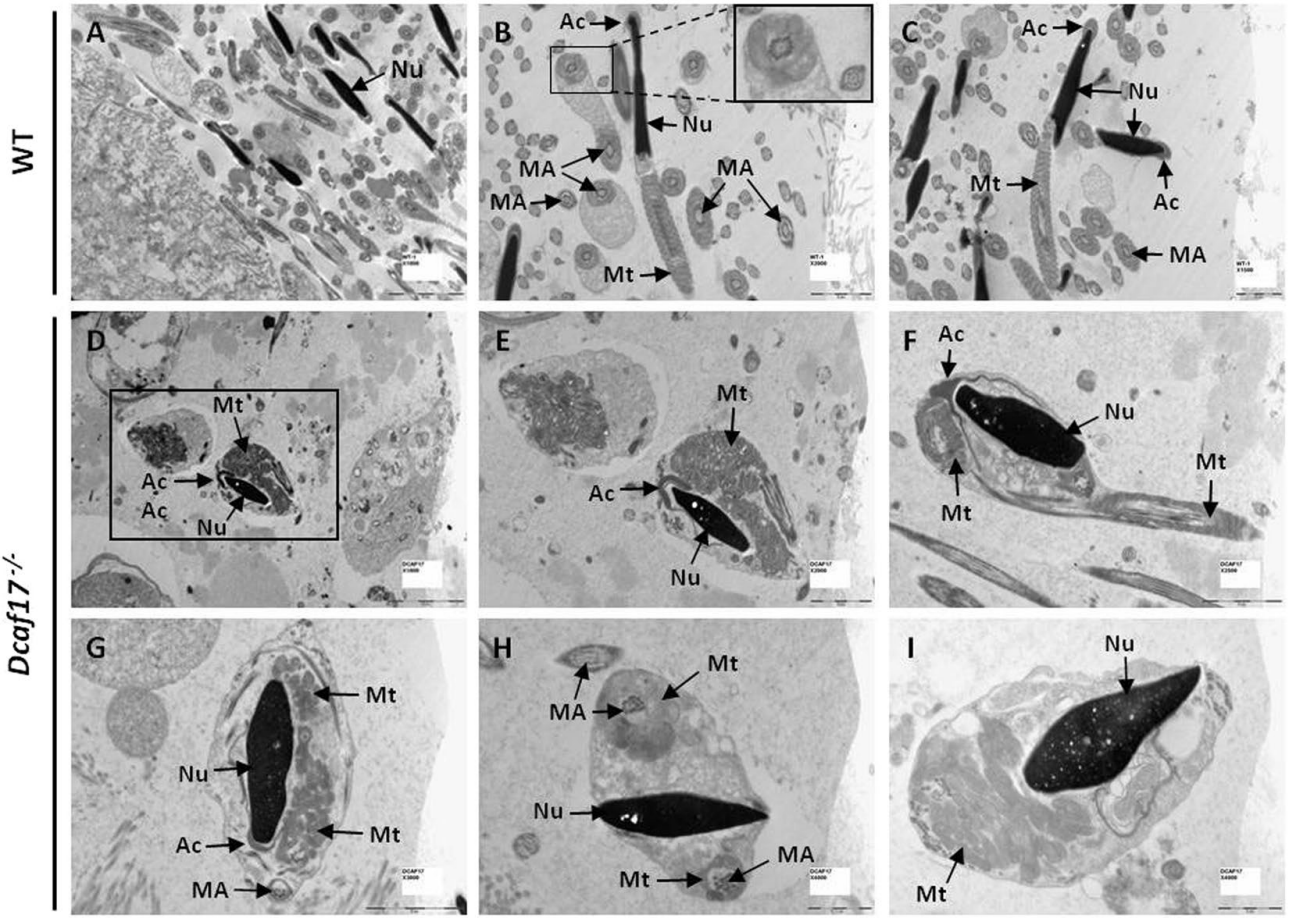
Transmission electron microscopy (TEM) of cauda epididymides from 8 weeks old WT (**A–C**) and *Dcaf17* ^*−/−*^ (**D–I**) adult mice. Representative TEM images of WT cauda epididymis (**A–C**) show spermatozoa with typical elongated nucleus (Nu) with homogeneously condensed chromatin. The acrosome (Ac) in WT sperm is covering the anterior portion of the head and is tightly attached to the nucleus through the acrosome-acroplaxome complex. The mid-piece of WT sperm show spirally arranged mitochondria (Mt) that are enclosed by well-defined mitochondrial sheath. WT sperm tail sections show the typical “9 + 2” pattern of the microtubular axoneme (MA). TEM images of *Dcaf17*^*−/−*^ cauda epididymis (**D–I**) show abnormal sperm with misshaped head containing defective nuclear (Nu) chromatin condensation, malformed and detached acrosome (Ac), disorganized mitochondria (Mt) trapped inside large cytoplasmic droplets and ectopic localization of the microtubular axoneme (MA). Inset image in panel B is enlarged image of squared region of image B. Image E is a higher magnification image of square region shown in the image D. Scale bar are shown at the bottom right corner to each image. Scale bar: A, D 5 µm; B, C, E, F, and G 2 µm; H and I 1 µm.

Further, analyzing the ultrathin section of cauda epididymides from WT and *Dcaf17*^*−/−*^ adult mice using TEM showed dramatic differences between the WT and *Dcaf17*^*−/−*^ sperm ultrastructure (Fig. 5). Almost all the WT sperm examined displayed normal head, mid-piece and tail structures (Fig. 5A–C). In the WT sperm, the head contained homogeneously condensed chromatin within the nucleus as shown by the density of staining observed in TEM images of WT sperm (Fig. 5A–C). The acrosome cap in the WT sperm was tightly associated with the nucleus through the acrosome-acroplaxome complex and covered the anterior portion of the head (Fig. 5A–C). The mid-piece of WT sperm possessed well-defined mitochondrial sheath enclosing mitochondria arranged in a spiral pattern around the outer dense fibers (ODFs) and the typical “9 + 2” pattern of the axonemal microtubules (Fig. 5B,C). Unlike the WT sperm, the *Dcaf17*^*−/−*^ sperm exhibited multiple ultrastructural abnormalities including misshaped head, defective nuclear chromatin condensation, detached acrosome, disorganized mitochondria trapped inside large cytoplasmic droplets and ectopic localization of the flagella (Fig. 5D–I). The head and mid-piece of the mutant sperm were often surrounded by asymmetric mass of residual cytoplasmic droplet in which the mitochondria and the components of flagellar structures were scattered and could not be assembled correctly (Fig. 5D–I). The presence of residual cytoplasm may damage tight associations of the acrosome with the nuclear membrane by expanding sub-acrosomal and perinuclear space that resulted in the detachment of the acrosome (Fig. 5D–G). The nuclei of *Dcaf17* KO sperm had chromatin with defective compaction as evident from uneven density and punctate texture of the nuclei (Fig. 5E–I). The mutant spermatozoa often showed partially formed or completely lacking connecting piece (Fig. 5F,G). Sperm from *Dcaf17*^*−/−*^ mice displayed disrupted mitochondrial sheath with disorganized ODFs, and axoneme microtubules (Fig. 5E–I). The “9 + 2” microtubular structure was disturbed in the mutant sperm (Fig. 5H). These abnormalities are similar to the defects seen in oligoasthenoteratozoospermia, a human infertility disorder characterized by low number of sperm, poor sperm motility, and abnormal sperm shapes with deformed round heads, distorted nuclei, abnormal acrosomes, and malformed mitochondrial sheaths^32,33^.

### *Dcaf17*^*−/−*^ mice have abnormal spermatogenesis

**Figure 6:**
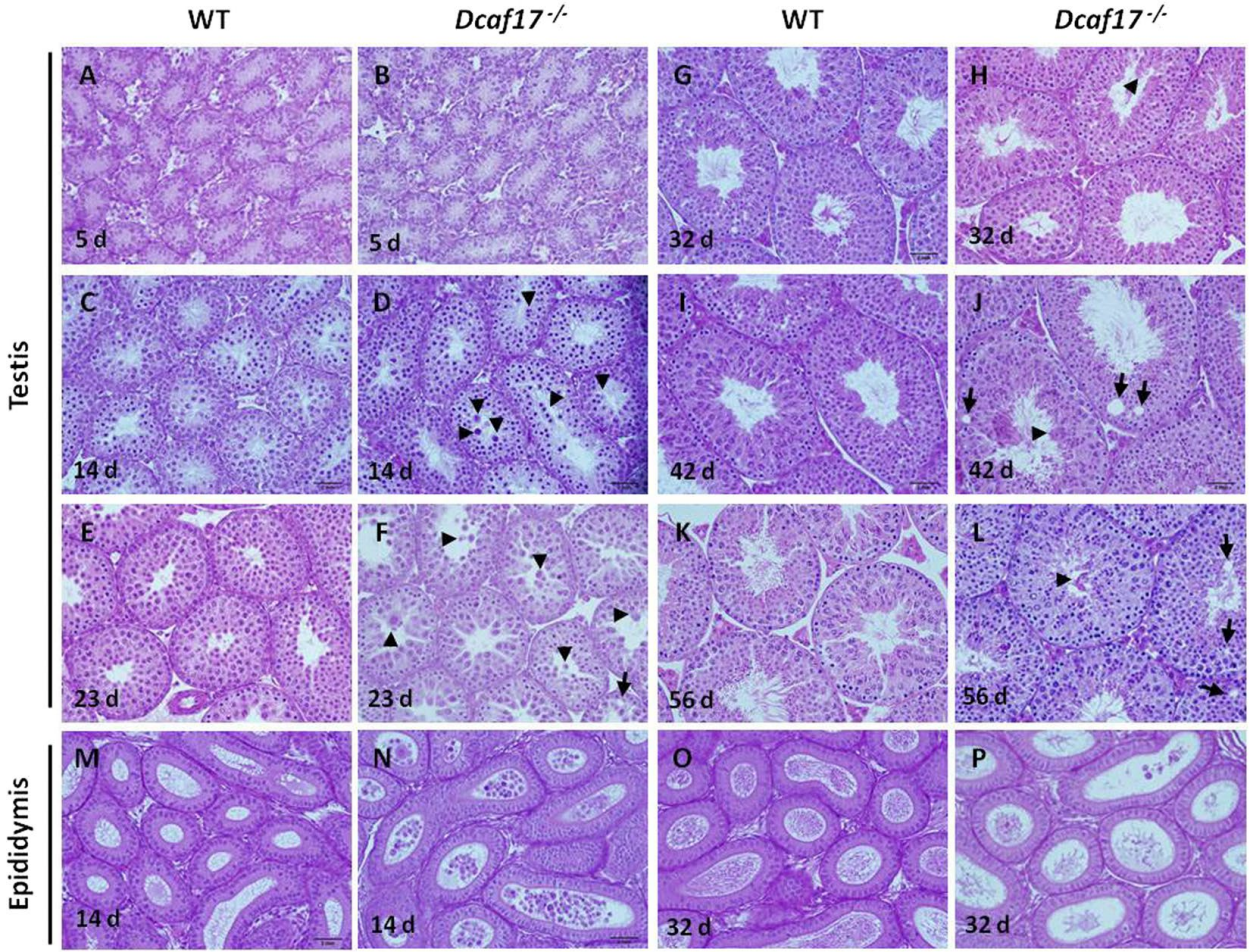
Histology of testes and cauda epididymides from WT and *Dcaf17* ^*−/−*^ mice at different ages. Sections (5 µm thick) of testes and cauda epididymes from WT and *Dcaf17*^*−/−*^ mice at different ages were stained with Hematoxylin and Eosin. Testes of WT (**A**) and *Dcaf17*^*−/−*^ (**B**) mice at 5 days postpartum (dpp) show normal Sertoli cells with no apparent morphological or histological differences. At 14 dpp, normal spermatocytes are seen in the WT (**C**) and *Dcaf17*^*−/−*^ (**D**) testes sections. Unlike WT testis at 14 dpp, the lumen of *Dcaf17*^*−/−*^ seminiferous tubules (ST) (**D**) show large shaded cells (arrow heads). Spermatocytes at most advanced stage and round spermatids are observed in the ST of the WT (**E**) and *Dcaf17*^*−/−*^ (**F**) testes at 23 dpp. The lumen of ST in *Dcaf17*^*−/−*^ testis at 23 dpp (**F**) shows giant, multinucleated and prematurely sloughed cells (arrow heads). Epithelial vacuoles (arrow) are also seen in the *Dcaf17*^*−/−*^ testis at 23 dpp. At 32 dpp, the WT testis (**G**) shows numerous elongating and elongated spermatids with normal morphology and chromatin condensation. *Dcaf17*^*−/−*^ testis at 32 dpp (**H**) shows fewer elongating and elongated spermatids with abnormal shape and chromatin condensation. Testes from 42 dpp (**I**) and 56 dpp (**K**) old WT mice show a complete cycle of spermatogenesis with seminiferous tubules depicting various stages of highly organized spermatogenic cells. Testes of *Dcaf17*^*−/−*^ mice at 42 dpp (**J**) and 56 dpp (**L**) show abnormal spermatogenesis with sever defects in post-meiotic stages of spermatogenesis. Vacuoles (arrows) are observed in the epithelium of some ST of the *Dcaf17*^*−/−*^ testis (**J**,**L**). The lumen of cauda epididymis (**M**) from sexually immature (14 dpp) WT mouse is devoid of any degenerated cells. In *Dcaf17*^*−/−*^ mouse at 14 dpp, the lumen of cauda epididymis shows numerous prematurely degenerated oval shaped cells of various size. Sexually mature cauda epididymis of WT mouse (32 dpp) has lumen (**O**) full of normal mature sperm. Prematurely degenerated spermatogenic cells of various size and few abnormal sperm are observed in the lumen of *Dcaf17*^*−/−*^ mouse cauda epididymis (**P**) at 32 dpp. Magnification 400X. Scale bar 2 mm.

To determine the onset of spermatogenic defects in *Dcaf17* KO mice, histological analyses were performed on developing testes from WT and *Dcaf17*^*−/−*^ mice at 5, 14, 23, 32, 42 and 56 dpp (Fig. 6). Analysis of testis sections by bright field microscopy at each age showed no differences in the thickness of the seminiferous epithelium between wild-type and *Dcaf17* mutant mice testes, indicating that lumen formation by the seminiferous epithelia was unaffected in the absence of DCAF17. At 5 dpp, the seminiferous tubules of WT and *Dcaf17*^*−/−*^ testes contained normal Sertoli cells and spermatogonia with no obvious histological differences in seminiferous tubular size and the population of germ cell types (Fig. 6A,B). At 14 dpp, normal spermatocytes were observed in the seminiferous tubules of WT and *Dcaf17*^*−/−*^ testes (Fig. 6C,D). However, some of the *Dcaf17*^*−/−*^ seminiferous tubules showed abnormal pachytene spermatocytes with less condensed nuclei that were detached from seminiferous epithelium (Fig. 6D). Male mice at 14 days of age were sexually immature; hence, there is no production of mature sperm in the testis and consequently, the cauda epididymal lumen of WT mice were devoid of any spermatogenic cells (Fig. 6M). Interestingly, the cauda epididymal lumen of *Dcaf17*^*−/−*^ mice at 14 dpp showed numerous degenerated spermatogenic round cells of various sizes (Fig. 6N). This indicates a defective process of spermatogenesis in *Dcaf17* KO mice. Spermatocytes at most advanced stage and round spermatids were observed in the seminiferous tubules of WT testes at 23 dpp (Fig. 6E). Although the seminiferous tubules of *Dcaf17*^*−/−*^ testes at 23 dpp showed advanced spermatocytes and round spermatids, many of the tubules contained giant cells, multinucleated cells, vacuoles and sloughed round spermatids (Fig. 6F). At 32 dpp, elongating and elongated spermatids were abundant with normal morphology and chromatin condensation in WT testis (Fig. 6G). In *Dcaf17* KO testis at 32 dpp, elongating and elongated spermatids were abnormal in shape and chromatin condensation (Fig. 6H). At 42 and 56 dpp, complete cycle of spermatogenesis within seminiferous tubules depicting various stages of highly organized spermatogenic cells was observed in WT testis sections (Fig. 6I,K). The *Dcaf17* KO testes at similar ages showed vacuoles, abnormal spermatogenesis with severe defects in both meiotic spermatocytes and post-meiotic spermatids (Fig. 6J,L). Spermiogenesis defects in the mutant mice were evident from reduced number of elongated spermatids and spermatozoa, abnormal-appearing spermatids, sloughed germ cells and the accumulation of residual bodies in the lumen of *Dcaf17*^*−/−*^ testis and epididymides (Fig. 6D,F,H,J,L,N,P).

**Figure 7:**
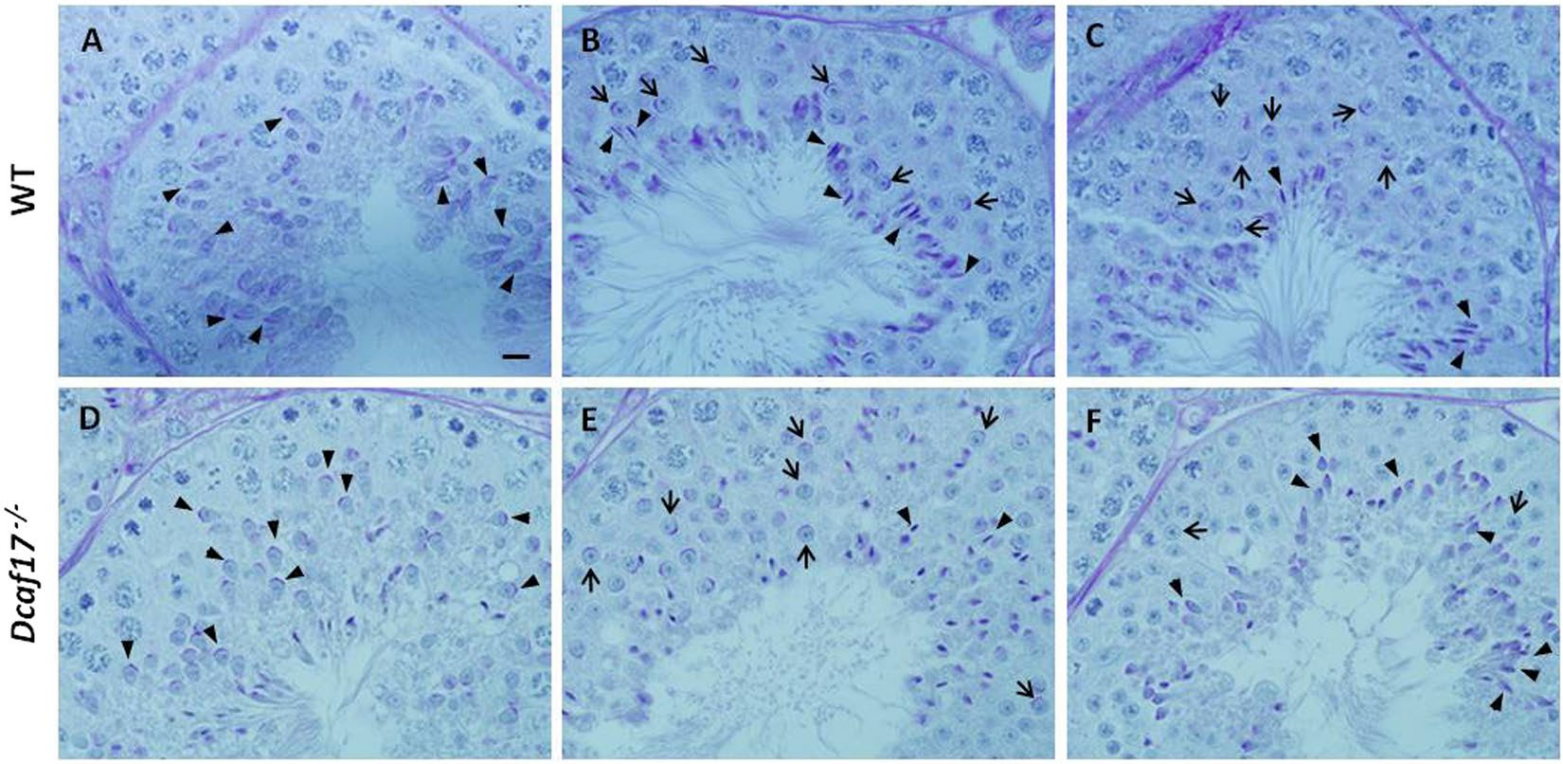
Periodic acid-Schiff (PAS) staining of testes sections from 8 weeks old WT and *Dcaf17*^*−/−*^ mice. To visualize the glycoproteins/acrosomes (violet) and nuclei (blue), the testis sections from WT (**A–C**) and *Dcaf17*^*−/−*^ (**D–F**) mice were stained with PAS-stain and hematoxylin counter stain. WT testis sections (**A–C**) show normal spermatogenesis with well-organized stages of germ cell development, round spermatids with PAS-positive normal acrosomal caps (arrows), elongating and elongated spermatids (arrow heads) and chromatin condensation. *Dcaf17*^*−/−*^ testis sections (**D–F**) show defective spermatogenesis with abnormal acrosomal caps (arrows), distorted elongated spermatids (arrows heads) and chromatin condensation. Magnification – 1000X. Scale bar: 10 µm.

H & E staining of *Dcaf17*^*−/−*^ testes at different ages revealed predominantly severe defects in post-meiotic spermatids (Fig. 6). To investigate spermiogenesis defects in detail, Periodic acid–Schiff (PAS) staining of the adult WT and *Dcaf17*^*−/−*^ testes sections was performed. WT testis displayed normal acrosomal caps, elongated spermatids and chromatin condensation (Fig. 7A–C). However, *Dcaf17*^*−/−*^ testis showed abnormal morphology of elongating and elongated spermatids with defective acrosome and chromatin condensation (Fig. 7D–F).

### Disruption of *Dcaf17* leads to increased germ cells apoptosis

**Figure 8:**
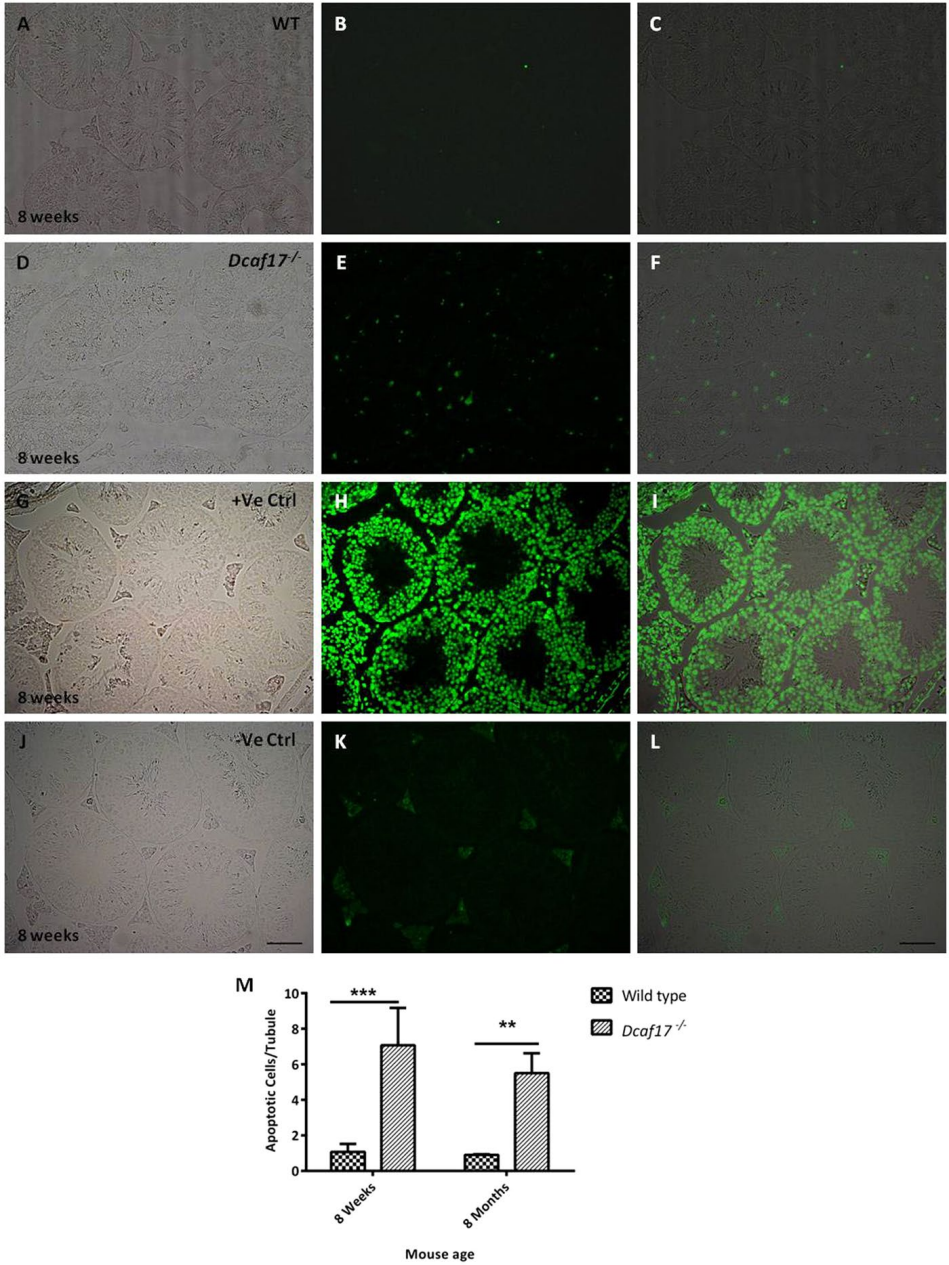
TUNEL staining of testes sections from 8 weeks old WT and *Dcaf17*^*−/−*^ mice to assess germ cell apoptosis. Testes sections of 8 weeks old WT (**A–C**) and *Dcaf17*^*−/−*^ (**D–F**) mice were subjected to TUNEL assay, which detects fragmented DNA in the apoptotic germ cells (green). WT testis section (**A–C**) shows fewer TUNEL-positive cells (green) (**B**,**C**) compare to *Dcaf17*^*−/−*^ testis section (**E**,**F**). Image H is positive control for TUNEL assay where the testis section was treated with DNaseI enzyme to generate fragmented genomic DNA. Image K is a negative control where testis section was treated only with labelling solution without terminal transferase. Images A,D,G and J are bright field images of respective TUNEL stained fluorescence images B, E, H, and K. Images C,F,I,L are merged images of respective bright field and fluorescence images. Magnification 200X. Scale Bar (**J**): 50 µm. The number of TUNEL-positive cells (green) per tubule in the testes sections of WT and *Dcaf17*^*−/−*^ mice aged 8 weeks and 8 months were plotted in the graph (**M**). The Histogram (**M**) represents mean ± SEM of 3 animals analyzed for TUNEL assay from each strain. Asterisks indicate statistical significance (p < 0.001 at 8 weeks and P < 0.01 at 8 month). Data were analyzed on Graphpad prism 5 using two-way ANOVA technique followed by post hoc Bonferroni’s multiple comparison test.

Due to observed reduction in sperm counts and presence of sloughed germs cells in the *Dcaf17* KO epididymides, apoptosis was assessed in the seminiferous epithelium of *Dcaf17* KO mice and compared with that of WT mice. TUNEL assay on the testes sections of 8 weeks (Fig. 8) and 8 months (Fig. S6) old WT and *Dcaf17*^*−/−*^ mice was performed. The results revealed an overall increase in the average number of TUNEL-positive apoptotic cells per seminiferous tubule in *Dcaf17*^*−/−*^ testis sections in comparison to that of wild type (P < 0.01value) (Fig. 8D–F,M). Interestingly, there was a variation in the number of TUNEL-positive cells in different *Dcaf17*^*−/−*^ tubule sections, suggesting that apoptosis is more pronounced at specific stages of germ cell development (Fig. 8D–F,M). In contrast, WT testes showed fewer number of TUNEL-positive cells per seminiferous tubule within the WT testis sections (Fig. 8A–C,M). Together, these findings suggest that DCAF17 deficiency causing impaired spermatogenesis accompanied by high rate of apoptosis.

### Meiotic progression is not affected in *Dcaf17* KO mice

**Figure 9:**
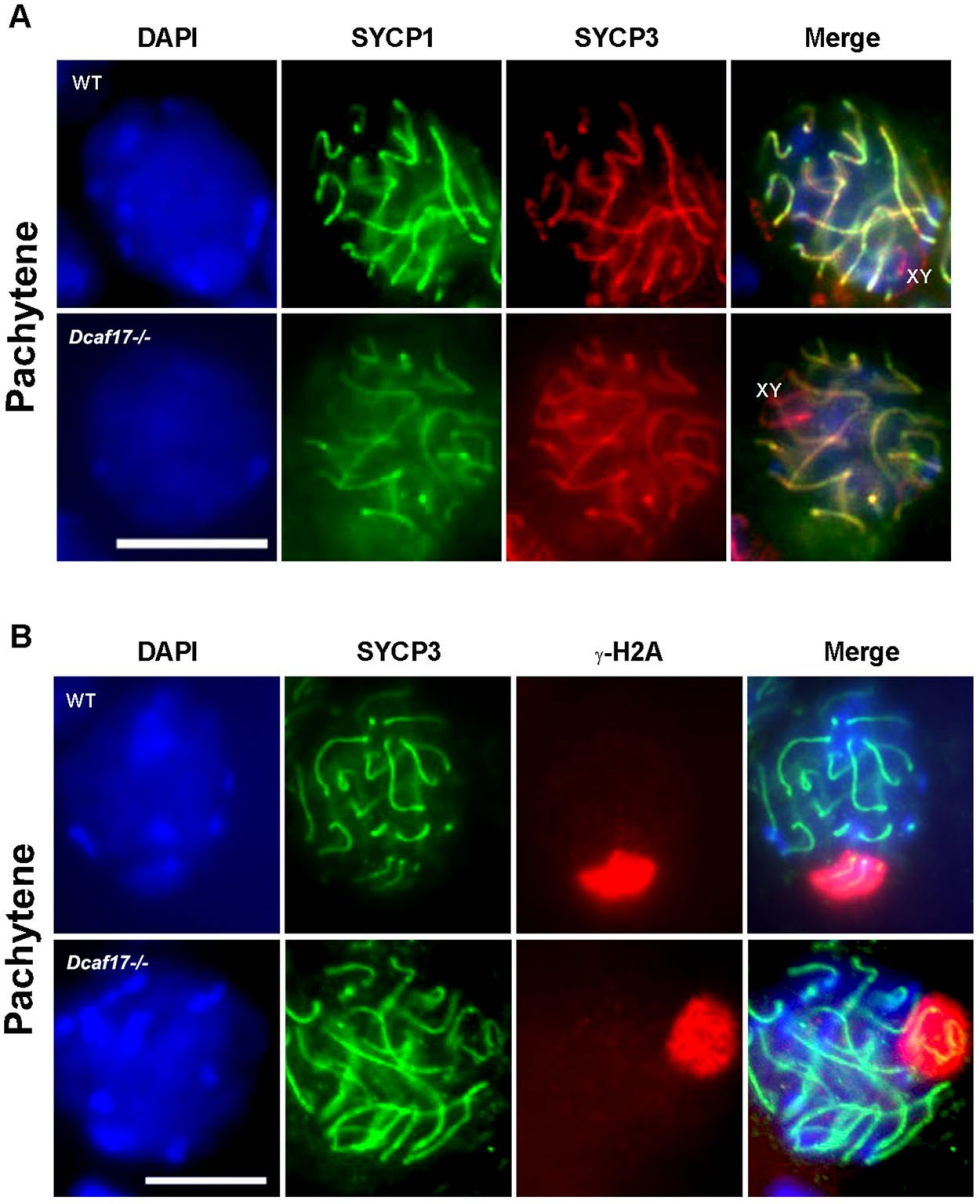
Immunofluorescence analysis of meiotic spermatocytes of 8 weeks old WT and *Dcaf17*^*−/−*^ testes. Immunostaining of the synaptonemal complex with SYCP1 (green) and SYCP3 (red) antibodies in pachytene spermatocytes of WT and *Dcaf17* mutant mice (Panel A). Immunofluorescnece staining of γ-H2AX (red) and the lateral element of the synaptonemal complex, SYCP3 (Green) in pachytene spermatocytes of WT and *Dcaf17*^*−/−*^ mice (Panel B). Spermatocytes with synaptic X and Y chromosomes (green) around which γ-H2AX (red) was normally distributed in WT and *Dcaf17*^*−/−*^ strains. Nucleus is counter stained with DAPI (blue). Magnification 1000X. Scale bar: 10 µm.

To determine whether the *Dcaf17* mutant spermatocytes progress properly through meiosis, we analyzed the expression and localization patterns of meiotic markers by immunolabeling of squashed preparation from WT and *Dcaf17*^*−/−*^ adult mice testes. We used antibodies against axial element, SYCP3 (also called SCP3) and transverse filaments, SYCP1 (also called SCP1) to examine the formation of synaptonemal complex and phosphorylated histone H2AFX (γH2AX) antibody to detect DNA double strand breaks (DSBs) before the synaptonemal complex is fully established and the unsynapsed X and Y chromosomes during the process of meiosis^34–36^. The labeling patterns for SYCP3 and SYCP1 proteins, in *Dcaf17*^*−/−*^ spermatocytes were indistinguishable from that in WT littermate spermatocytes (Fig. 9A). Thus synapsis formation appears to be normal in *Dcaf17* mutant germ cells. During meiosis, DNA DSBs occur in early prophase and are then repaired as germ cells reach the pachytene stage. To examine the effect of *Dcaf17* disruption on DNA DSBs and XY body formation during meiosis, double immunostaining of SYCP3 and γH2AX was performed. During leptotene and early zygotene, γH2AX spreads in the nucleus in response to DNA DSBs. In mid-pachytene to late diplotene spermatocytes, γH2AX was located on the sex chromosomes as a result of meiotic sex chromosome inactivation. The localization of γH2AX was identical in leptotene/zygotene and pachytene of both *Dcaf17* mutants and WT mice (Fig. 9B), suggesting that similar to WT spermatocytes, DSBs occurred and were repaired in the *Dcaf17* mutant spermatocytes. Together, our data suggests that DCAF17 does not play significant role in meiotic events during spermatogenesis.

### Defective manchette formation and nuclear compaction during spermiogenesis in *Dcaf17*^*−/−*^ mice

**Figure 10:**
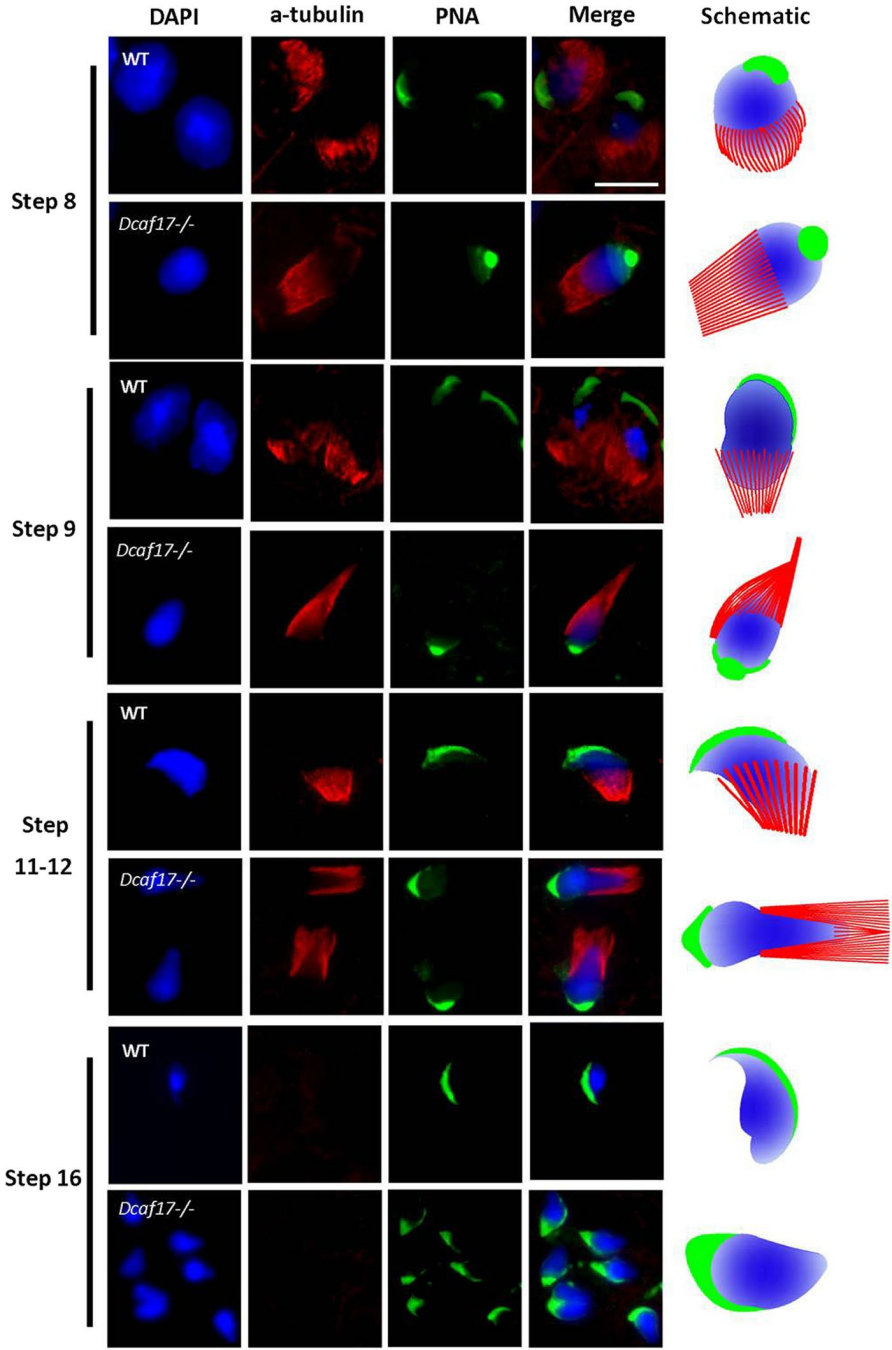
Manchette and acrosome formation based on α-tubulin and PNA immunofluorescence staining of testes squash preparations from WT and *Dcaf17*^*−/−*^ mice. Testes squash preparations from WT and *Dcaf17*^*−/−*^ testes from adult mice were stained using antibodies against α-tubulin (red) and lectin PNA (green) to analyze manchette and acrosome formation, respectively. Nucleus was stained with DAPI (blue). Representative fluorescence images of microtubule (red), acrosome (green) and nucleus (blue) staining and corresponding merge images of post-meiotic male germ cells at different steps of spermiogenesis are shown for both the genotypes (WT and *Dcaf17*^*−/−*^) at similar stages. Different steps of sperimioenesis are depicted on the basis of nuclear and acrosome staining. WT post-meiotic germ cells show normal microtubule bundles (manchette) assembly (red), acrosome morphogenesis (green) and nuclear (blue) elongation during spermiogenesis. In the *Dcaf17* mutant spermiogenic cells, the organization of microtubule bundles (red) around the nucleus (blue) is disrupted resulting in abnormal manchette formation, defective nuclear (blue) condensation and elongation, and abnormal acrosome (green) morphogenesis. Ectopic microtubules, defective acrosome and abnormal nuclear head shaping are noticeable in *Dcaf17*^*−/−*^ post-meiotic germ cells. Magnification 1000X. Scale bar: 10 µm. Schematic representations of microtubule bundles (red) assembly, acrosome (green) formation in relation to the nucleus (blue) shaping during spermiogenesis in wild-type and *Dcaf17* KO spermatids are shown.

Disruption of *Dcaf17* in mice caused male infertility due to abnormality in sperm morphogenesis. Aberrations in the sperm head and flagellar structures are often associated with malformation of the manchette, a transient microtubule structure that appears around the nucleus of elongating spermatid and plays crucial roles both in head shaping and cargo transport to the tail region in developing spermatids^10,37–40^. The timing of manchette formation is very precise and coincides with the process of nuclear shaping and flagellum development. The sperm head and tail deformities observed in *Dcaf17* mutant mice might arise from defects in the formation or function of manchette. To examine abnormalities in manchette formation, acrosome morphogenesis and nuclear elongation during spermiogenesis in the *Dcaf17* KO testis, immunofluorescence staining was performed on WT and *Dcaf17*^*−/−*^ testes squash preparations using anti α-tubulin and FITC conjugated PNA lectin antibodies and DAPI to identify manchette, acrosome and nucleus, respectively. No significant morphological differences were observed between early round spermatids from spermiogenesis steps 1 to 7 of WT and *Dcaf17* mutant mice (Data not shown). In WT testis, the manchette formation begins at step 8 of spermiogenesis, when elongation of the round spermatid starts by nucleus polarization and change of nucleus shape from spherical to slightly elongated (Fig. 10). During the normal manchette biogenesis, the microtubule bundles assemble at the perinuclear ring surrounding posterior region of early elongating spermatid nucleus (Fig. 10)^10,40^. As the spermiogenesis progresses, the perinuclear ring and manchette move towards distal part of the sperm head forming a slanted conical-shaped manchette (Fig. 10). The manchette moves toward the tail neck region and disappears by the end of spermiogenesis. While assembly of manchettes in *Dcaf17*^*−/−*^ testis appeared to start at the correct time, their organization and nuclear elongation were affected (Fig. 10). In the *Dcaf17* mutant testis, abnormal manchette organization and nuclear elongation started from step 8 onwards and continued throughout the subsequent processes of spermiogenesis (Fig. 10). The manchettes of *Dcaf17*^*−/−*^ spermatids were more elongated and showed abnormal distal movement along the nuclear surface (Fig. 10). Throughout the elongation phase of *Dcaf17*^*−/−*^ spermatids, the perinuclear ring of the manchette continued to narrow that resulted in an unusual bulb-like nucleus structure with an enlarged spherical anterior region and a long tapered posterior region (Fig. 10).

Co-immunostaining of WT and *Dcaf17* mutant testes squash preparations by PNA-FITC to stain acrosome revealed that there were no significant differences in acrosome biogenesis during Golgi phase (steps 1–3) and cap phase (steps 4–7) of spermiogenesis between two genotypes, which is consistent with PAS staining of testes sections (Fig. 7). During the acrosome phase (steps 8–12) in WT testis, when nuclear condensation and elongation take place and manchette formed around the posterior region of nucleus, the acrosomal cap further elongated and produced characteristic crescent-shaped acrosome. However, the *Dcaf17*^*−/−*^ spermatids during acrosome phase showed impaired acrosome morphogenesis that was manifested by failure of acrosome cap extension (Fig. 10).

**Figure 11:**
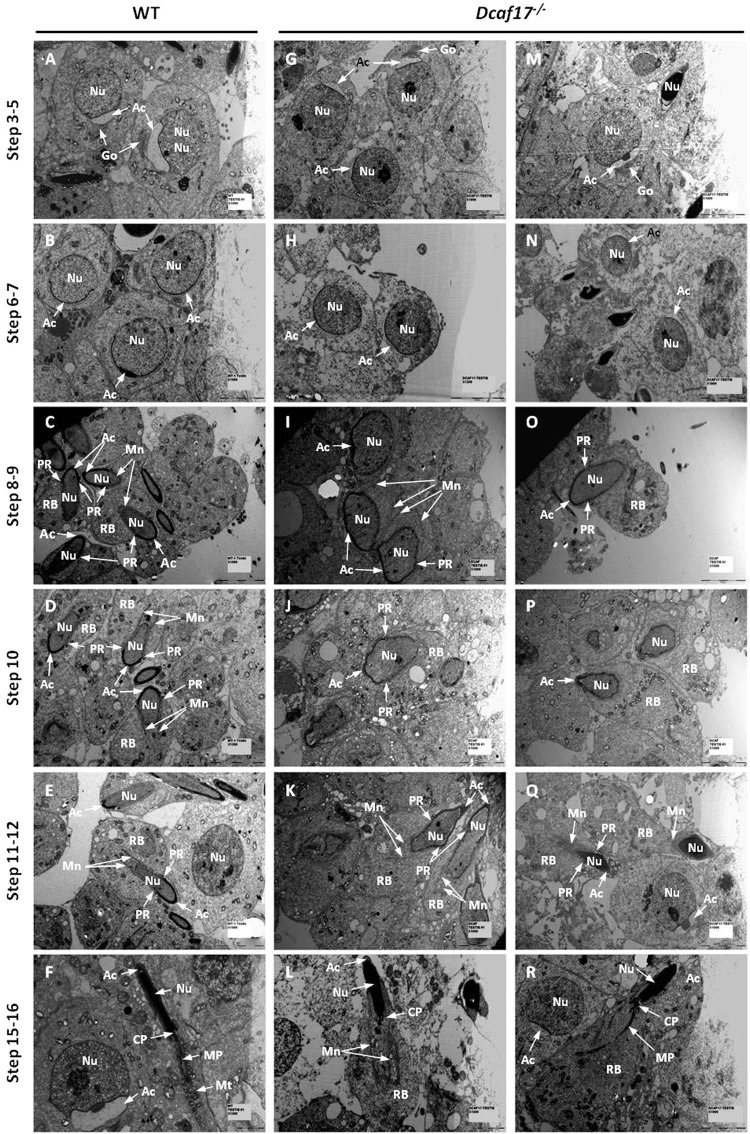
Testicular ultrastructure of WT and *Dcaf17*^*−/−*^ adult mice by transmission electron microscopy (TEM). Representative TEM images of 8 weeks old WT (**A–F**) and *Dcaf17*^*−/−*^ (**G–R**) mice testes ultrathin sections displaying different steps of spermiogenesis. Spermatids in step 3–5 (**A**,**G**,**M**), step 6–7 (**B**,**H**,**N**), step 8–9 (**C**,**I**,**O**), step 10 (**D,J,P**), step 11–12 (**E**,**K**,**Q**) and step 15–16 (**F**,**L**,**R**) are represented. In WT (**A**) and *Dcaf17*^*−/−*^ (**G**,**M**) testes, round spermatids during steps 3–5(**A**,**G**,**M**) show acrosome (Ac) attached with spherical nucleus (Nu) at one end. Some round spermatids show Golgi (Go) apparatus located close to acrosome (Ac). During steps 6 and 7 of spermiogenesis in WT (**B**) and *Dcaf17*^*−/−*^ (**H**,**N**) testes, the acrosome (Ac) flattens and grows to form a cap covering approximately half of the nuclear (Nu) surface. During steps 8–12 of WT spermiogenesis, the spermatid nucleus (Nu) starts to elongate and the acrosome (Ac) extends along the nuclear envelope (**C**,**D**,**E**). The manchette (Mn) in WT elongating spermatids (**C**,**D**,**E**) extends from perinuclear ring (PR) into residual body (RB) and attached to posterior region of nuclear membrane. The *Dcaf17*^*−/−*^ elongating spermatids in steps 8–12 (**I–K** and **O–Q**) show abnormal nuclear elongation and asymmetric extension of the acrosome (Ac) that is associated with distorted nucleus (Nu). The perinuclear ring (PR) in the elongated spermatids of *Dcaf17*^*−/−*^ mutant (**I–K** and **O–Q**) is abnormally positioned and microtubule bundles of manchette (Mn) are severely disorganized along the posterior surface of the deformed nucleus (Nu). WT elongated spermatids in steps 15–16 show elongated nucleus (Nu), a midpiece (MP) region containing uniformly distributed mitochondria (Mt) and connecting piece (CP) joining the midpiece region of the tail to the head of maturing spermatozoa. The manchette (Mn) and residual body (RB) gradually disappear during the maturation steps 15–16 in WT. Elongated spermatids at steps 15–16 in *Dcaf17*^*−/−*^ testis (L,R) show oval shaped nucleus (Nu), abnormal manchette (Mn) (**L**) and residual body (RB). Scale bars: (**A–E)**, (**G–K)** and (**M–R)** 5 µm; F and L 2 µm.

To characterize the abnormalities in *Dcaf17*^*−/−*^ spermiogenesis at the ultrastructural level, TEM analyses of testes from WT and *Dcaf17* mutant mice were carried out. No obvious differences in the ultrastructure of spermatids at Golgi phase and cap phase (steps 1–7) were observed between WT and *Dcaf17* mutant mice (Fig. 11A,B,G,H,M,N). However, the elongating and elongated spermatids from *Dcaf17*^*−/−*^ testis were most affected and showed various abnormalities in acrosome, nucleus, perinuclear ring and manchette. While elongating and elongated spermatids in WT seminiferous tubules showed typical flattened and elongated nuclei, the *Dcaf17*^*−/−*^ spermatids at corresponding developmental steps showed abnormally shaped nuclei (Fig. 11C–F,I–L,O–R). The WT elongating and elongated spermatids showed normal appearing condensed chromatin (Fig. 11D–F). In contrast, chromatin compaction was defective in the elongating and elongated spermatids of *Dcaf17*^*−/−*^ mice (Fig. 11J–L,P–R). In differentiating WT spermatids, the acrosome is attached to nuclear envelop symmetrically covering the anterior part of the nucleus (Fig. 11B–F). Although the acrosome in most of the *Dcaf17*^*−/−*^ elongating spermatids was attached to the nuclear envelope, its distribution was asymmetric and aligned the deformed nucleus (Fig. 11I–L,O–R). The manchette in normal spermatids appeared as a parallel arrangement of microtubule bundles extending from a perinuclear ring into a distal cytoplasm and attached to posterior nuclear membrane (Fig. 11C–E). In *Dcaf17*^*−/−*^ spermatids, the microtubule bundles of manchette was severely disorganized (Fig. 11I–L,O–R). Furthermore, *Dcaf17*^*−/−*^ spermatids displayed abnormal positioning of the perinuclear ring (Fig. 11I–L,O–Q). During normal spermiogenesis, the manchette moves toward the tail neck region around steps 13–14 and gradually removed in subsequent steps^10^ (Fig. 11C–F). Interestingly, disruption of *Dcaf17* resulted in delayed removal of manchette and retention of residual cytoplasm (Fig. 11L,R). Collectively, these data suggests the role of DCAF17 in microtubule manchette organization during spermiogenesis.

## Discussion

Spermiogenesis is a complex metamorphosis process of male germ cell where the round haploid spermatids undergo a series of remarkable cytoskeletal and nuclear changes to generate mature elongated spermatozoa. Multiple and tightly regulated cellular and molecular events are orchestrated in the generation of normal mature spermatozoa^6,8,41^. Disruption in underlying mechanisms could cause abnormal sperm formation, which often leads to male infertility^8,10^. In the present study, we demonstrated that *Dcaf17* gene is highly expressed in testis with low level of expression in other organs like brain, liver, pancreas and skin. Targeted disruption of the *Dcaf17* gene revealed that it is not essential for normal embryonic development and resulted in male infertility without other identifiable anatomical or behavioral abnormalities. Despite normal female KO mice fertility, the male infertility phenotype of the *Dcaf17* KO mice is attributed to the severe malformation and disorganization of specialized structures of the spermatozoa.

E3 ubiquitin ligases play essential roles in regulating every stage of spermatogenesis starting from spermatogonia to differentiated spermatozoa^12,15,42^. Large number of E3 ligases have been identified that are expressed during mouse spermatogenesis with many of them are shown to be expressed highly or specifically in the testis^43^. Similarly, *Cul4A* and *Cul4B* have been shown to express highly in mouse testis^23,25^. The CUL4A and CUL4B exhibited complementary expression patterns in adult mouse testis, where CUL4A primarily expressed in spermatocytes during meiosis, while CUL4B was highly expressed in Sertoli cells, spermatogonia and spermatids^24^. Differential expression patterns of CUL4A and CUL4B in testis at different space and time, highlight key, non-overlapping roles for each protein in testicular development and spermatogenesis. Our findings showed predominant abundance of *Dcaf17* mRNA levels in adult mouse testis, implying a crucial role of DCAF17 in testicular function. Further, the *Dcaf17* transcripts were gradually increased with the mouse age from low at neonatal age to highest at puberty and remained constant. At 5 dpp, when there were mainly Sertoli cells and spermatogonia within the testis, the *Dcaf17* mRNA levels were low. Low levels of *Dcaf17* transcripts at neonatal age suggest that DCAF17 may not have crucial role during early mitotic phase of spermatogenesis. However, this need to be confirm by experimental analysis. High expression of *Dcaf17* gene at the age of puberty may be due to a pivotal role of DCAF17 during later stages of spermatogenesis.

Male infertility phenotype observed in *Dcaf17* KO mice can be due to defective reproductive physiology, sex organ development or sexual behavior. Normal mating behavior of *Dcaf17* mutant mice and comparable anatomical structure, size and weight of *Dcaf17*^*−/−*^ testes suggested that the infertility is not due to defective reproductive physiology, sex organ development or sexual behavior. However, the infertility phenotype in *Dcaf17* KO mice is caused mainly because of the defective germ cell development as shown by testicular histology, sperm analyses and TEM. The onset of spermatogenic defect in *Dcaf17*^*−/−*^ mice occurred at 14 days after birth when pachytene spermatocytes occur. Early disruption of spermatogenesis in the *Dcaf17*^*−/−*^ testis was evidenced by premature sloughing of spermatogenic cells from seminiferous epithelium into the lumen of seminiferous tubules. The TUNEL data indicated that *Dcaf17* deficiency caused germ cell apoptosis that is exacerbated with animal age and resulted in accumulation of sloughed premature germ cells within the KO mice epididymides.

The most remarkable phenotype observed in *Dcaf17*^*−/−*^ mice was an acute defect in post-meiotic germ cell development, resulting in increased number of dysmorphic and immotile sperm. During the normal spermiogenesis process, the round haploid spermatids undergo a series of extensive morphological changes to generate mature functional spermatozoa. The major changes during this process include acrosome biogenesis, acroplaxome and manchette formation, chromatin condensation, nucleus elongation, and assembly and elongation of flagellum^8,44^. In the final step of spermiation, residual cytoplasm is eliminated, specialized intercellular adhesion structures are removed and the spermatid is detached from the Sertoli cell and released into the lumen of the seminiferous tubule^2,8^. Defects in these processes lead to a lack of functional spermatozoa, which is a major cause of male infertility in the human population^45,46^. We showed that *Dcaf17*^*−/−*^ mice produced malformed spermatozoa due to defective spermiogenesis. This defect was histologically characterized by defective elongating and elongated spermatids and degenerative germ cells that were observed in the lumen of seminiferous tubules and epididymides. Aberrant sperm head and flagellar structures are often attributed to malformed manchette development during spermiogenesis^8,10,40^. Alpha-tubulin localization at different stages of spermatid development in *Dcaf17*^*−/−*^ testis revealed abnormal microtubule assembly, suggesting the role of DCAF17 in manchette formation. Further, electron microscopic analysis of testis ultrathin sections confirmed the aberrant manchette formation during *Dcaf17*^*−/−*^ spermiogenesis. In addition to manchette malformation, the ultrastructural studies also showed defects in nucleus shaping, chromatin condensation and asymmetric localization of acrosome. Along with the prematurely degenerated round sloughed germ cells, the cauda epididymis lumen of *Dcaf17*^*−/−*^ mice showed very low number of malformed globular shaped sperm which were rarely motile. Immunofluorescence staining of micotchondria, acrosome and nucleus of *Dcaf17*^*−/−*^ cauda epididymal sperm showed ectopic localization of mitochondrial sheath and acrosome with less condensed nucleus.

In several studies, it was shown that the CUL4 ubiquitin ligases (CUL4A and CUL4B) play essential roles in male germ cell development. Both *Cul4A* and *Cul4B* mutant male mice were infertile^22–25^. Although CUL4A and CUL4B share sequence and structural similarities, both the proteins play diverse roles in spermatogenesis^23–25,47^. Disruption of *Cul4A* in mice resulted in male infertility due to abnormal meiotic progression, whereas disruption of *Cul4B* in mice caused male infertility because of aberrant post-meiotic sperm development^22–25^. Macroscopic features of *Cul4A* KO mice testes showed two fold reductions in the size and the weight of the *Cul4A*^*−/−*^ testis compared to that of wild type^25^. Whereas, *Cul4B* KO mice had no significant effect on the size and weight of the testis^23^. Similar to *Cul4B* KO mice, deletion of *Dcaf17* did not affect the testicular size and weight, indicating that disruption of *Dcaf17* in mice do not have major effect on the development of testis at macroscopic level. Drastic reduction in sperm production and sperm motility due to defective spermatogenesis have been shown in both the, *Cul4A* and *Cul4B* KO mice^23,25^. Comparable to *Cul4A* and *Cul4B* KO mice, deletion of *Dcaf17* also disrupted normal spermatogenesis causing significant reduction in sperm number and motility. Epididymal histology of *Cul4A* and *Cul4B* KO mice showed to have very few sperm with mostly abnormal morphology. In addition to few sperm, epididymis lumen of *Cul4A* and *Cul4B* KO mice have also been shown to contain degenerated cells^23,25^. Likewise, histological examination of *Dcaf17*^*−/−*^ epdidymis in this study also showed that the lumen of *Dcaf17* KO epididymis contained very few, mostly abnormal sperm along with round sloughed cells of varying sizes. Deletion of *Dcaf17* in mice resulted in formation of vacuoles in the seminiferous epithelium, significant increase in apoptotic cells, remarkable reduction in elongated spermatids and spermatozoa, and release of degenerated germ cells in the lumen of the seminiferous tubules. Similar testicular phenotypes have also been reported in the testes of *Cul4A* and *Cul4B* KO mice, independently^23,25^. Investigation of sperm development in *Cul4A* deficient mice by two independent groups revealed that CUL4A is required for the regulation of meiotic progression through controlling meiotic recombination and double stranded break^24,25^. Deletion of *Cul4A* in mice have been shown to disrupt meiosis process in the testis, causing considerable reduction in the number of round spermatids^25^. Unlike *Cul4A* KO mice, the *Cul4B* KO mice testis showed significant decrease in elongating and elongated spermatids due to increased apoptosis during cap phase and acrosome phase^23^. The *Cul4A* and *Cul4B* KO mice have been shown to have severe defects in acrosome formation, mitochondrial sheath and chromatin condensation during sperm development at ultrastructural level^22–24^. Equally, fluorescence microscopic and ultrastructural analysis of *Dcaf17* KO testis and spermatozoa showed severe defects in normal acrosome formation, manchette organization, chromatin condensation and nuclear elongation during spermiogenesis. The severe infertility phenotype observed in *Dcaf17* KO male mice implicates indispensible function(s) of DCAF17 proteins during normal sperm development.

DCAF17 is a member of a DCAF family proteins that function as receptor protein for different Cullin4-based E3 ligase complexes and provides substrate specificity to the E3 ligase complex for protein ubiquitination^27^. Ubiquitination plays very important roles in regulation of different aspects of normal sperm development and testicular functions^15,48–50^. The CUL4A and CUL4B function as a scaffold protein for Cullin4-based E3 ligase complex assembly^26^. Due to the comparable similarities in the testicular phenotypes of *Cul4A*, *Cul4B* and *Dcaf17* KO mice, we speculate that the function(s) of CUL4A, CUL4B and DCAF17 proteins may be interlinked for the regulation of normal spermatogenesis via ubiquitin mediated protein homeostasis. However, it is needed to be confirmed by appropriate experimental analyses.

In conclusion, our studies on *Dcaf17* KO mice successfully characterized the male infertility phenotype caused due to *Dcaf17* deletion and demonstrated the importance of DCAF17 protein in maintaining normal spermiogenesis. It has also provided an ideal model to further investigate the molecular mechanisms of DCAF17, E3 ligase complex components and their target proteins in sperm development and male fertility.

## Materials and Methods

### Animals

Conditional *Dcaf17* knockout (KO) mice were generated by inGenious Targeting Laboratory, Inc. (Ronkonkoma, NY, USA). The CMV-*Cre* mice (B6.C-Tg(CMV-*cre*)1Cgn/J) were obtained from the Jackson Laboratory (Bar Harbor, ME, USA). Mice were housed in the animal facility at King Faisal Specialist Hospital and Research Centre, Riyadh, Saudi Arabia, under controlled conditions with 12 hours light/dark cycles and free access to feed and water. All the experiments and animal procedures were approved by the Animal Care and Use Committee of the King Faisal Specialist Hospital and Research Centre (Protocol RAC# 210-0006), and executed according to institutional and international guidelines for the care of experimental animals.

### Targeted disruption of *Dcaf17* gene

Disruption of *Dcaf17* gene in the mouse genome was carried out using gene targeting approach. Briefly, a 10.46 kb fragment containing exon 4 of *Dcaf17* gene from *Dcaf17*-containing C57BL/6 BAC clone (RP23: 359E23) was subcloned to construct the targeting vector (pSP72 backbone). The FRT/loxP flanked *Neo* cassette was inserted upstream of exon 4 in intron 3–4 in an opposite direction in relation to the target gene. The single loxP site was inserted downstream of the exon 4 in intron 4–5 sequence. The target region was 930 bp long and included exon 4 of *Dcaf17* gene. The region was designed to have a short homology arm (SA) extending 1.4 kb upstream of the LoxP/FRT-flanked pGK-gb2 *Neo* cassette and a long homology arm (LA) extending 8.06 kb to the 3′-end of the single lox P located downstream of the exon 4. The targeting construct was linearized by *NotI* restriction digestion and transfected into the BA1 (C57BL/6 × 129/SvEv) mouse embryonic stem cells (mESCs). The targeted mESC clones were selected for G418 antibiotic resistance. Positive mESC clones were screened by PCR using A1 or A2 forward primers and UNI reverse primer to confirm the short homology arm integration (Figs 2A and S2B). The PCR-positive mESC clones were further confirmed by Southern blotting (Fig. S2C) using the specific probes shown in the Figures S2A and B. Targeted mESC clones containing floxed exon 4 of *Dcaf17* gene were microinjected into C57BL/6 blastocysts to produce chimeric mice. Chimeras with a high percentage agouti coat color were mated to wild-type C57BL/6 mice to generate F1 heterozygous offspring (*Dcaf17*^*wt/fl)*^). The *Dcaf17*^*wt/fl*^ mice were back-crossed to generate homozygous mice for *Dcaf17*^*fl/fl*^, on a C57BL/6 background. The breeding was continued to establish the founder line. Genomic DNA from tail biopsies of offspring was genotyped for the presence of the *Dcaf17*^*fl/fl*^ allele by PCR using LOX1 and LAN1 primers, and sequenced by SDL2 and LOX1 primers to confirm LoxP site retention (Figs 2A and S2B). The *Dcaf17*^*fl/fl*^ floxed mice were mated with CMV-*Cre* mice (B6.C-Tg(CMV-cre)1Cgn/J) to remove the neomycin cassette and *Dcaf17* exon 4. *Cre*-mediated deletion of *Dcaf17* exon 4 was confirmed by performing PCR using WSS-F and WSS-R primers on tail DNA samples collected from the F1 pups as shown in Fig. 2B. As a further confirmation, RNA was extracted from tail snips and RT-PCR with primers Dcaf17-3F and Dcaf17-5R was used to discriminate between the various genotypes (Fig. 2C). *Dcaf17*^+/−^ mice were crossed to generate *Dcaf17*^*−/−*^ homozygous mutants.

### Genomic DNA extraction and PCR Genotyping

Genomic DNA was isolated from tail biopsies using Gentra Puregene Mouse Tail Kit (Qiagen, Hilden, Germany), according to manufacturer’s instructions. Genotype analysis, to confirm excision of *Dcaf17* exon 4, was performed by routine PCR. The primers used for genotyping are listed in Table 2. PCR conditions were 94 °C for 15 minutes, followed by 35 cycles at 94 °C for 30 seconds, 58 °C for 30 seconds, and 72 °C for 1 minute, followed by a 10 minute extension at 72 °C. The PCR products were separated on 1% agarose gel prepared in 1X TAE (Tris-acetate-EDTA) buffer (40 mM Tris acetate and 1 mM EDTA) containing ethidium bromide at the final concentration of 0.5 µg/ml. Agarose gel electrophoresis was carried our using Owl Horizontal Electrophoresis running chamber (Thermo Scientific, USA) and PowerPac 300 (BIO-RAD Laboratories, Hercules, CA, USA) power supply. Agarose gel images were taken using ImageQuant LAS 4000 (GE Healthcare Life Sciences, Pittsburgh,PA, USA) gel imaging system under standard UV exposure of 1/8 seconds. Adobe Photoshop software (Adobe Inc., San Jose, CA, USA) and Microsoft PowerPoint software were used to assemble images into figures. No post-acquisition modifications were made to the original images.

**Table 2:** List of primers used in this study.

Primer Name	Primer Sequence (5′-3′)	Purpose
Sp6	GAGTGCACCATATGGACATATTGTC	Sequencing targeting vector
T7	TAATGCAGGTTAACCTGGCTTATCG	Sequencing targeting vector
N1	TGCGAGGCCAGAGGCCACTTGTGTAG	Sequencing targeting vector
N2	TTCCTCGTGCTTTACGGTATCG	Sequencing targeting vector
CARF3	TGCTCTAGTTGAAGACTTGAGG	Sequencing targeting vector
A2	ACA CGT TCA TGT ATG AGC CCA G	ESC screening
A1	ACACTCTGAGGTAGTTTATAGTCG	ESC screening
UNI	AGCGCATCGCCTTCTATCGCCTTC	ESC screening
LOX1	TTGCATACTCACATCCATTTGG	F1 and floxed mice genotyping/LoxP site retention
SDL2	TGCTCTAGTTGAAGACTTGAGG	F1 and floxed mice genotyping/LoxP site retention
LAN1	CCAGAGGCCACTTGTGTAGC	F1 genotyping/LoxP site retention
WSS-F	TTGCTCCTCTTTCCCTTCTG	*Dcaf17 KO mice genotyping*
WSS-R	CAAATTGCATACTCACATCCATT	*Dcaf17 KO mice genotyping*
Dcaf17-3F	GTTGCATCAGAGCCAAGAAA	*RT-PCR*
Dcaf17-5R	TTGTTCTGAGCCGACTTCAC	*RT-PCR*
Dcaf17-F	GTTGTACCTCGCAGTGTTCC	*qRT-PCR*
Dcaf17-R	GATGGCTTGGAAGCTGTAGA	*qRT-PCR*
18 s rRNA-F	CTCTTTCGAGGCCCTGTAAT	*qRT-PCR*
18 s rRNA-R	CTCCCAAGATCCAACTACGA	*qRT-PCR*

### Mating behavior and fertility test

Sexually mature (8–10 weeks old) male mice (n = 3 to 5) of each genotypes, WT and *Dcaf17*^*−/−*^, were mated individually with sexually mature (8–10 weeks old) female mice of WT or *Dcaf17*^*−/−*^ genotype (n = 3 to 5) in a controlled breeding experiment for the period of 2–6 months. Females were observed for the presence of vaginal plugs and signs of pregnancy. Litter numbers and pups produced by contorlled breeding were recorded.

### Sperm count, motility and morphology

The cauda epididymides from 8 weeks and 8 months old WT and *Dcaf17*^*−/−*^ mice (n = 3) were dissected and collected in 1 ml of EmbryoMax Human Tubal Fluid (HTF) medium (Millipore, MA, USA) pre warmed at 37 °C. The collected cauda epididymides were incised using fine scissors and sperms were allowed to swim out by incubating for 15–30 minutes at 37 °C under 5% CO_2_. Ten μl of sperm suspension medium was transferred to the hemocytometer and the total number of sperm was determined using a brightfield microscope (MICROMASTER, Fisher Scientific, Waltham, MA, USA). The total numbers of sperm were counted and the average was calculated from 5 microscopic fields per caudal epididymis. Sperm motility was evaluated immediately by placing a 10 μl drop of sperm suspension on hemocytometer and counting the sperm under a brightfield at 400× magnification. Sperm motility presented as percentage of motile sperm of total sperm number.

To study the sperm morphology, sperm smears were prepared by pipetting 20 µl of cauda epididymides sperm suspension on glass slides. After drying the slide, the sperm smear was fixed with 2% paraformaldehyde (PFA) by incubating at room temperature for 10–15 minutes, washed three times with 1X PBS and stained with Diff-Quick stain (Shandon™ Kwik-Diff™ Stains, ThermoFisher Scientific, Waltham, MA, USA) according to the manufacturer’s protocol. Diff-Quick stained slides were observed under optical microscope (Olympus IX71, Olympus, Center Valley, PA, USA). Digital images were captured using OLYMPUS DP72 microscope camera (Olympus, Center Valley, PA, USA) and OLYMPUS cellSens Entry 1.6 imaging software (Olympus, Center Valley, PA, USA). Adobe Photoshop software (Adobe Inc., San Jose, CA, USA) and Microsoft PowerPoint software were used to assemble images into figures. No post-acquisition modifications were made to the original images.

### Histology

For the evaluation of spermatogenesis, histological analyses were performed on the testes of WT and *Dcaf17* KO mice at different postnatal ages (5, 14, 23, 32, 42 and 56 days) as well as on epididymides of sexually mature (8 weeks old) mice (n = 3). The tissues were collected and directly fixed in 4% paraformaldehyde (PFA) or in Bouin’s fixative for overnight at 4 °C. Fixed tissues were washed with distilled water and dehydrated in a series of 70–100% ethanol solutions. Dehydrated tissues were embedded in paraffin and 5 µm thick sections were prepared on glass slides using microtome (Leica RM2155, Leica Biosystems, Buffalo Grove, IL, USA). Paraffin embedded tissue sections were deparaffinized, rehydrated and stained with hematoxylin and eosin (H&E) or periodic acid-Schiff (PAS) staining according to standard protocols^51,52^. Stained testis and epididymis sections were observed at room temperature using Olympus BX53 (Olympus, Center Valley, PA, USA) optical microscope. Digital images were captured using OLYMPUS DP72 microscope camera (Olympus, Center Valley, PA, USA) and OLYMPUS cellSens Entry 1.6 imaging software (Olympus, Center Valley, PA, USA). Adobe Photoshop software (Adobe Inc., San Jose, CA, USA) and Microsoft PowerPoint software were used to assemble images into figures. No post-acquisition modifications were made to the original images.

### Testes squash preparation

Squash preparation of testicular samples was performed as previously described^53^. Briefly, the testes of adult WT and *Dcaf17*^*−/−*^ mice were dissected and decapsulated by removing tunica albuginea. Seminiferous tubules of decapsulated testis were dispersed and fixed in freshly prepared 2% formaldehyde in phosphate-buffered saline (PBS) containing 0.05% Triton X-100 (Sigma, St. Louis, MO, USA) for 10 minutes at room temperature. Pieces (~1 mm) of seminiferous tubules were placed in a drop (~15 µl) of fixing solution on a clean glass slide. The pieces were gently minced with fine tip tweezers and were covered with glass cover slip. The cells were squashed by applying external pressure on coverslip. The slides were frozen by immersing in liquid nitrogen. The slides were immediately used for immunostaining or stored at −80 °C until required.

### Immunofluorescence

Evaluation of defects in sperm head and mid piece was carried out using MitoTracker, Peanut agglutinin (PNA) and 4′,6-Diamidino-2-Phenylindole, Dihydrochloride (DAPI) staining as described previously^24^. Briefly, the cauda epididymides were dissected from sexually mature (8–10 weeks old) WT and *Dcaf17*^*−/−*^ mice (n = 3 for each genotype) and sperm were allowed to swim out as described before. Aliquots of sperm suspension were transferred to 1.5 ml centrifuge tubes and centrifuged at 500 × g for 10 minutes at room temperature. The HTF medium was removed and the sperm were incubated with MitoTracker solution (Invitrogen, ThermoFisher Scientific, Waltham, MA, USA) at 37 °C for 30 minutes. Sperm were collected by centrifugation and then fixed with 4% PFA, permeabilized in 0.2% Triton X-100 (sigma-Aldrich, St. Louis, MO, USA) and a smear was prepared. Slides were then incubated with PNA-FITCI (GeneTex, Irvine, CA, USA) (1:1000 dilution in PBS) at room temperature for 45 minutes. Slides were washed and mounted with VECTASHIELD mounting medium containing DAPI (1.5 µg/ml) (Vector Laboratories, Burlingame, CA, USA).

For immunostaining of testes squash preparations, the slides were hydrated and washed in PBS. After washing in PBS, the slides were boiled for 20 min in 1× DakoCytomation Target Retrival Solution (pH 6.0) (Dako, Carpinteria, CA, USA) using a microwave oven for antigen unmasking. The slides were then washed three times in PBS for 5 min each and blocked for non-specific antibody binding by incubating with 10% normal goat serum (Gibco, ThermoFisher Scientific, Waltham, MA, USA) in PBS for 1 h at room temperature. After blocking, the slides were washed with PBS and incubated overnight at 4 °C with appropriate primary antibodies diluted in 0.5% BSA and 0.1% Triton X100 in PBS. The following antibodies were used: mouse monoclonal anti-SYCP3 (97672, Abcam, Cambridge, MA, USA) and rabbit polyclonal anti-SYCP1 (15090, Abcam, Cambridge, MA, USA) at 1:200 dilution; rabbit polyclonal phospho-Histon H2A.x (ser139) antibody (2577, Cell Signaling, Danvers, MA, USA) at 1:500 dilution or mouse monoclonal alpha-tubulin (DM1A) (3873, Cell signaling, Danvers, MA, USA) at 1:1000 dilution. Following the primary antibody incubation, the slides were washed with PBS (3 times 5 minutes each) and were further incubated in dark with appropriate secondary antibodies at room temperature for 1 hour. All secondary antibodies; FITC conjugated goat anti-rabbit-IgG (SC-2012), rodamin conjugated goat anti-rabbit-IgG (SC-2091), FITC conjugated goat anti-mouse-IgG (SC-2010), or rodamin conjugated goat anti-mouse-IgG (SC-2092) were purchased from Santa Cruz Biotechnology (Santa Cruz, CA, USA) and used at 1:300 dilution. After secondary antibody incubation, slides were washed in PBS, and mounted in VECTASHIELD mounting medium with DAPI (1.5 µg/ml) (Vector Laboratories, Inc., Burlingame, CA, USA). For sperm and testes immunofluorescence analyses, three mice of each genotype were used. From each mouse, three slides were prepared. All the samples were observed at room temperature using a Zeiss Axio imager Z2 epifluorescence microscope (Carl-Zeiss, Oberkochen, Germany) and digital images were captured with an Axiocam MRm microscope camera (Carl-Zeiss, Oberkochen, Germany) and AxioVision imaging software ZEN2011. Adobe Photoshop software (Adobe Inc., San Jose, CA, USA) and Microsoft PowerPoint software were used to assemble images into figures. No post-acquisition modifications were made to the original images.

### TUNEL assay

Terminal deoxynucleotidyl transferase (TdT) mediated dUTP-biotin nicked end-labeling (TUNEL) assay was performed by using *In Situ* Cell Death Detection kit (Roche Diagnostics, Basel, Switzerland) to detect DNA fragmentation in apoptotic cells according to manufacturer’s recommended protocol. Briefly, testis paraffin sections from 8 weeks and 8 months old WT and *Dcaf17*^*−/−*^ mice testes were deparaffinized with xylene, rehydrated in dilution series of ethanol, and permeabilized by incubating the sections with proteinase K (Qiagen, Hilden, Germany) solution (20 µg/ml in 10 mM Tris-HCL, pH 7.4) for 15–30 minutes at room temperature. Following permeabilization, the tissue sections were washed twice in PBS and incubated with TUNEL reaction mixture for 60 minutes at 37 °C in the dark. Positive controls were treated with 3 U/ml Dnase I (Roche Diagnostics, Basel, Switzerland) solution (prepared in 50 mM Tris-HCL, pH 7.5, 10 mM MgCl_2_ and 1 mg/ml BSA) for 10 minutes before TUNEL labeling, while negative controls were treated with labeling solution without TdT enzyme. After TUNEL mixture incubation, sections were washed three times (5 minutes each) with PBS, and mounted in VECTASHIELD mounting medium (Vector Laboratories, Inc., Burlingame, CA, USA). Three serial testicular sections (5 µm) each from three different mice of each WT and *Dcaf17*^*−/−*^ genotypes were used for TUNEL assay. All the samples were observed under 20× objective at room temperature using a Zeiss Axio imager Z2 epifluorescence microscope (Carl-Zeiss, Oberkochen, Germany) and digital images were captured with an Axiocam MRm microscope camera (Carl-Zeiss, Oberkochen, Germany) and AxioVision imaging software ZEN2011. All the fluorescence images were acquired under the same exposure time (190.5 ms). The average number of TUNEL-positive cells per tubule was determined by analyzing 100 seminiferous tubules per animal. Adobe Photoshop software (Adobe Inc., San Jose, CA) and Microsoft PowerPoint software were used to assemble images into figures. No post-acquisition modifications were made to the original images.

### Total RNA extraction and reverse transcription

To determine *Dcaf17* mRNA levels in different tissues and during testis development, adult WT mouse tissues (brain, liver, pancreas, skin and testis) and 5, 14, 23, 32, 42 and 56 days postpartum (dpp) testes were collected and snap frozen immediately by immersing in liquid nitrogen. Total RNA was extracted using QIAGEN RNeasy mini kit and then treated with DNase I according to the manufacturer’s protocol (Qiagen, Valencia, CA, USA). The RNA quality was checked by visualizing intact 28S and 18S RNA bands on agarose gel-electrophoresis using ethidium bromide staining. Quantification of total RNA was carried out using NanoDrop 2000c spectrophotometer (Thermo Scientific, ThermoFisher Scientific, Waltham, MA, USA). Reverse transcription was performed on 500 ng total RNA from each tissue using Superscript III Frist Strand Synthesis system (Life Technologies, ThermoFisher Scientific, Waltham, MA, USA) as per manufacturer’s protocol.

### Quantitative Real Time PCR (qRT-PCR)

The qRT-PCR was performed using 5 µl of 1:5 diluted cDNA products as templates from different tissues in a 20 µl of total reaction mixture for each cDNA sample. The qPCR reaction mixture contained 10ul, SYBER Green Supermix (1725271, BIO-RAD Laboratories, Hercules, CA, USA) and 1 µl of 100 nM of each forward and reverse primer. For each sample, a parallel reaction was done using 18 s rRNA primers as endogenous control. Quantitative RT-PCR was carried out using C1000 Touch thermal cycler (CFX96 Real-Time system, BIO-RAD Laboratories, Hercules, CA, USA). Reaction steps were 95 °C for 5 min, followed by 40 cycles of denaturation at 95 °C for 30 sec, primer annealing at 58 °C for 30 sec, and primer extension at 72 °C for 30 sec, followed by melting curve analysis (55 to 95 °C; in 0.5 °C increments) to verify specificity of amplicons. All samples were analyzed in triplicate and 18 s rRNA was used as internal control for data normalization, which were analyzed by the 2^−ΔCt^ method and reported as fold change. Primers used for this experiment are listed in Table 2.

### Transmission electron microscopy (TEM)

For the ultra-structural analysis, testes and epididymides of WT and *Dcaf17*^*−/−*^ adult mice were collected, cut roughly into 1 mm cubes and immediately fixed in 2.5% glutaraldehyde solution prepared in 0.1 M PBS (pH 7.4) for 1 hour at room temperature. After glutaraldehyde fixation, tissues were rinsed twice 5 minutes each with 0.1 M PBS and post-fixed in 1% Osmium Tetroxide (OsO_4_) for 1 hour at room temperature. After the OsO_4_ treatment, tissues were washed with distilled water twice for 5 minutes each and pre-stained in 2% uranyl acetate for 30 minutes at room temperature. Tissues were then washed with distilled water for 5 minutes, dehydrated in a series of gradient acetone and embedded in Epon resin (Poly/BED 812). Semi-thin sections (0.5–0.7 µm) were cut using LEICA EM UC6 ultra microtome (Leica Biosystems, Buffalo Grove, IL, USA), stained with toluidine blue and inspected under the bright field microscope. The relevant areas were chosen and ultrathin (50–70 nm) sections were prepared with a diamond knife using LEICA EM UC6 ultra microtome. Sections were picked up on Gilder hexagonal mesh copper grids (G200HH, TED PELLA, INC., Redding, CA, USA) and stained with uranyl acetate followed by lead citrate. Ultrathin sections were examined with JEM-1230 JEOL transmission electron microscope (JEOL USA Inc., Peabody, MA, USA) operated at 60 kV. Digital images were acquired using KeenView Soft Imaging System camera (ResAlta Research Technologies, Golden, CO, USA) and iTEM imaging software (ResAlta Research Technologies, Golden, CO, USA).

### Statistical Analysis

All data from sperm count, motility, TUNEL assay and qRT-PCR were analyzed using Graphpad prism 5 software and presented as the mean ± standard error of the mean (SEM). All the experiments were performed in triplicate and were repeated three times. For each experiment, 3 to 5 animals were used. A one-way ANOVA or two-Ways ANOVA followed by post hoc Bonferroni’s multiple or Tukey’s multiple comparisons tests were performed to compare the data. A *P-*value < 0.05 was considered statistically significant.

### Data Availability

All data generated or analyzed during this study are included in this published article and its supplementary information files.

## Electronic supplementary material

Dataset 1
