# Publisher Correction: Deletion of DDB1- and CUL4- associated factor-17 (*Dcaf17*) gene causes spermatogenesis defects and male infertility in mice

**DOI:** 10.1038/s41598-018-29836-2

**Published:** 2018-08-01

**Authors:** Asmaa Ali, Bhavesh V. Mistry, Hala A. Ahmed, Razan Abdulla, Hassan A. Amer, Abdelbary Prince, Anas M. Alazami, Fowzan S. Alkuraya, Abdullah Assiri

**Affiliations:** 1 Comparative Medicine Department, King Faisal Specialist Hospital and Research Centre, Riyadh, 11211 Saudi Arabia; 2 Department of Genetics, King Faisal Specialist Hospital and Research Centre, Riyadh, 11211 Saudi Arabia; 3 Department of Biochemistry and Molecular Biology, Faculty of Veterinary Medicine, Cairo University, Giza, 12613 Egypt; 4 College of Medicine, AlFaisal University, Riyadh, Saudi Arabia; 5 Institute for Research and Medical Consultations, Imam Abdulrahman Bin Faisal University, Dammam, Saudi Arabia

Correction to: *Scientific Reports* 10.1038/s41598-018-27379-0, published online 15 June 2018

In the original version of this Article, the authors Hala A. Ahmed, Hassan A. Amer, Anas M. Alazami and Fowzan S. Alkuraya were incorrectly indexed. This error has now been corrected.

