# Supplementary material for: Deletion of DDB1- and CUL4- associated factor-17 (Dcaf17) gene causes spermatogenesis defects and male infertility in mice
Source: Sci Rep. 2018 Jun 15;8:9202. doi: 10.1038/s41598-018-27379-0 (PMC6003934; doi:10.1038/s41598-018-27379-0)
Supplement: Supplementary file 1 — Dataset 1 [file 41598_2018_27379_MOESM1_ESM.pdf]

Supplementary Information

Deletion of DDB1- and CUL4- associated factor-17 (*Dcaf17)* gene causes spermatogenesis defects and male infertility in mice

Asmaa Ali[1#], Bhavesh V. Mistry[1#], Hala A. Ahmed[1], Razan Abdulla[1], Hassan A. Amer[3], Abdelbary Prince[3], Anas M. Alazami[2], Fowzan S. Alkuraya[2] and Abdullah Assiri[1,4,5*]

[1]Comparative Medicine Department, King Faisal Specialist Hospital and Research Centre, Riyadh, 11211, Saudi Arabia

[2]Department of Genetics, King Faisal Specialist Hospital and Research Centre, Riyadh, 11211, Saudi Arabia

[3] Department of Biochemistry and Molecular Biology, Faculty of Veterinary Medicine, Cairo University, Giza, 12613, Egypt

[4]College of Medicine, AlFaisal University, Riyadh, Saudi Arabia

[5]Institute for Research and Medical Consultations, Imam Abdulrahman Bin Faisal University, Dammam, Saudi Arabia

Methods

Southern Blot Analysis

Genomic DNA from conditional KO positive mES cells was digested with *NcoI* and separated by 0.8% agarose gel electrophoresis. The DNA was transferred to nylon membrane and probed with *Dcaf17* gene-specific probes using standard Southern blotting protocol[1,2].

Sequence of internal probe used for Southern blotting:

ACTGTTTAAGCCATGAGCAGCAATATTCAGAATTTAATGTTAATTTTTTTCTAGGTTGTTGTTTTTCCATAAAGCTATAATTTTT
TTTAACTATCACTCCCCCCCCCCCATAATTACTCTTTTCATAGAAACCACCTTTTTAAAAATTAGAAAGTTAGAAGCCTGGTTTT
TTTCCTCTTTAATTGAAAGCTGTAAATAATTGTGCCTTAAACCACTTAGAGCCTTTTAATCATTTTCTTGAGGGGAGATTAGA
GAGATGATAGGTTTCTTAATTGAGAAAGATTGAATATACCAATTCAAACTCCCAAAATATAGAGCAGTCCTGAAAAAATAAA
GTTAACCTACTCTTATTCCATAAACACTTAAGTGATTTTGTTTTTATTTGTGGTGACAACATTTTTTATGAAGAACTCAGAGGA
GCACTGCATTATTATATGAATGCAAAACTACTTTAAAATGAATACTCTGTTTATTTTGTTTTGATTCTGAAGTAATGTATGAAC
TGCCACTTTTGGTTTAAGAAAAAAGTTCAAACCTGTAAGATACTTGTTTTAAATGTCTCTGTAATGGGCTAAATAGGAATAGT
AGATATTTTAAAACTTTAAAATCTACATGAAATGACACATAAGTCATGTAGCATAGAAGCATTCATGTCATGGCCT

Sequence of external probe used for Southern blotting:

TGATTCTAGCAGTGGCAGTCTGGACATTCTCTTACACCATTCTCATGATGCCCATCCTCTGCCTTGCAGTGTTAACAGTGACT
CTTAGGCACCGTTTCATACCCAGGGTGTTGCCTTCCCAAGTCAGAGACAGTTATTTTGATTTTAAAAAGTTCAGTCAGAGTAA
ATGCCATTGATTGCTGTCGCAGTGATAAATGATGCAGGTGTTCTTCACCGTTCTGTTGCAGAGGGTGGCACCCACAGTTAGA
GTCACATGAGATTGCTTTCACTCCTGTGACATTCAGTTTGATTTAAACCTTTGAGTTACTGAGAGAATACCAGAGGTGAATAA
AATGTCATCCTGGAAATTGTTTCTCTTCTCAGTAGTTGAACTTTAAAGTTCGTCAGTTAGAAAGAAAAAAAGACATCTATGAC
TGCATCTCAGTAGTTGTCAGAGTAGAGACTAGCTGGAGGTGGGGTAGGGGCCTGTAGCGTCAGCCCAGGGAAGCTGAGGC
AAGGGGGATTGCTTGAGGACAGCTTGGGCAAGATTGT

Supplementary Figures

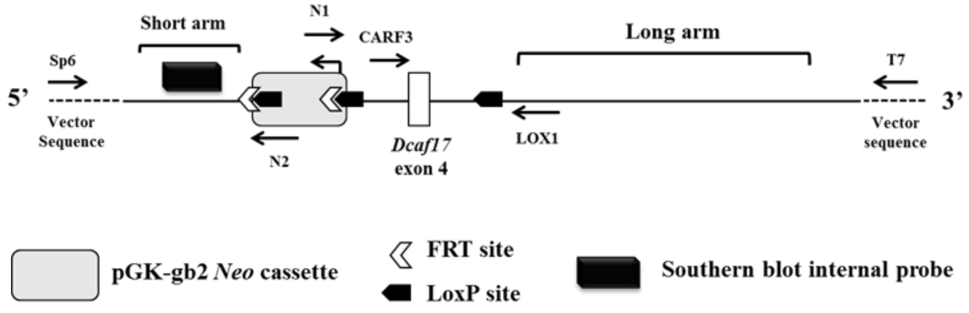

Figure S1: Schematic representation for *Dcaf17* gene targeting vector. A DNA fragment of 10.46 kb was subcloned to construct targeting vector containing a 1.46 kb short homology arm (SA) upstream of the LoxP/FRT-flanked pGK-gb2 Neo cassette and an 8.06 kb long homology arm (LA) downstream of the only lox P site located outside the Neo cassette. The exon 4 of *Dcaf17* gene is shown with white box. The LoxP/FRT-Neo cassette is inserted upstream of exon 4 in intron 3-4 in an opposite direction regarding the targeting gene. The single Lox P site is inserted downstream of exon 4 in intron 4-5 sequence. Primers used for sequencing and PCR confirmation are indicated with arrows.

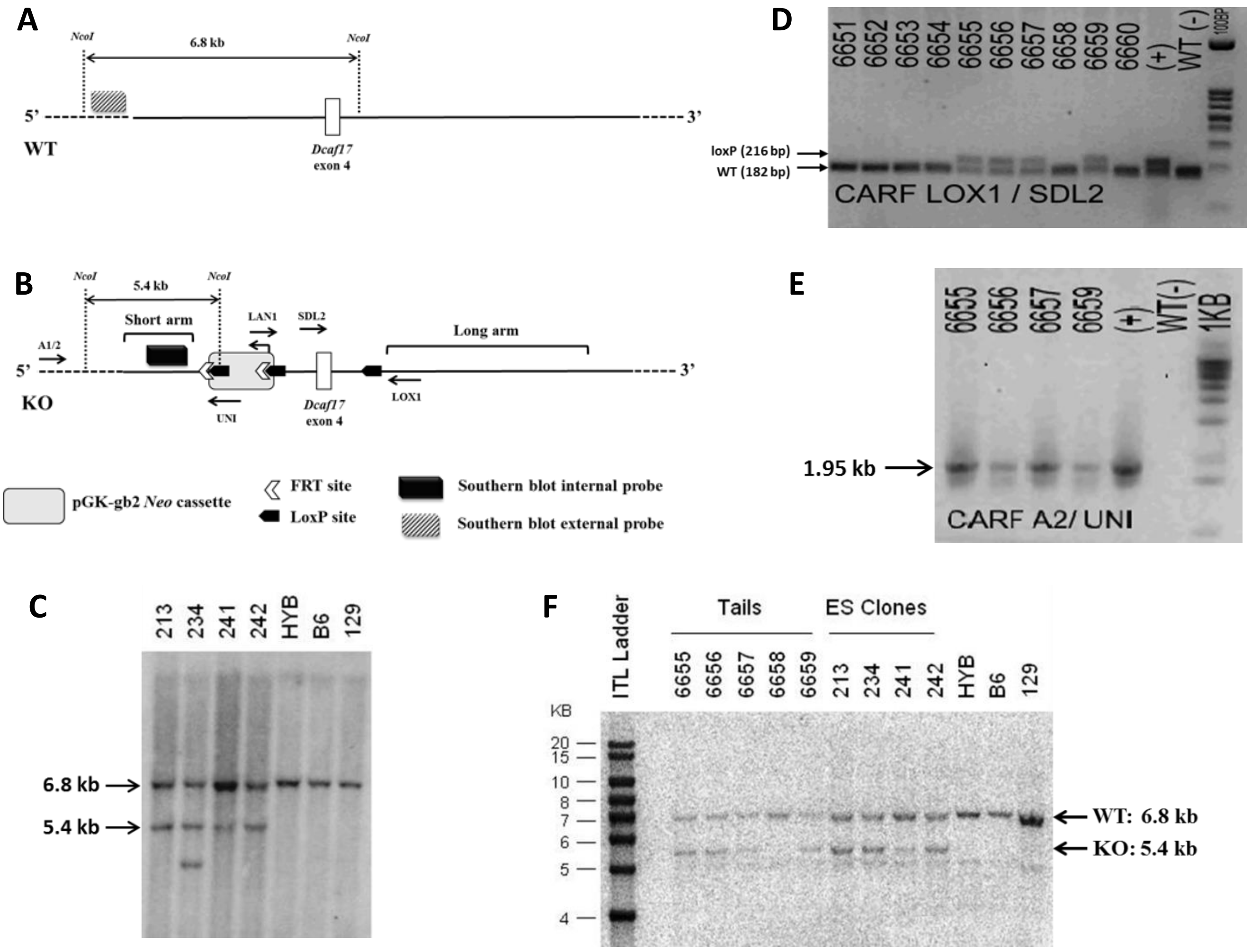

Figure S2: *Dcaf17* conditional knockout gene targeting screening by PCR and Southern blotting. (A) Schematic representation of the wild type allele of *Dcaf17* gene targeting region. Exon 4 is shown in white box. Positions of *NcoI* restriction enzyme near the Exon 4 are indicated. Size of the restriction fragment is shown. Position of the specific external probe used for Southern blotting is indicated by black stripped rectangle. (B) Schematic representation of conditional targeting region of *Dcaf17* gene. The exon 4 of *Dcaf17* gene is shown with white box. The *LoxP/FRT-Neo* cassette is inserted upstream of exon 4 in intron 3-4 in an opposite direction regarding the targeting gene. The single *LoxP* site is inserted downstream of exon 4 in intron 4-5 sequence. Primers used for PCR confirmation are indicated with arrows. Short and long homology arms are shown. Position of the specific internal probe used for Southern blotting is indicated by solid black rectangle. Positions of *NcoI* restriction enzyme near the exon 4 and size of the restriction fragment are shown. (C) Confirmatory Southern blot image of *Dcaf17* conditional KO positive mESC clones (213, 234, 241 and 242) that were positive by PCR screening. DNA from C57Bl/6 (B6), 129/SvEv (129), and BA1 (C57Bl/6 x 129/SvEv) (Hybrid) mouse strains were used as wild type controls. (D) Agarose gel image of PCR products amplified using LOX1 and SDL2 primers to screen the F1 mice colony of *Dcaf17* conditional KO for *LoxP* retention. The F1 mice with *LoxP* site retention showed 216 bp PCR amplicon, whereas the F1 mice with no *LoxP* site showed 182 bp PCR amplicon. The expanded *Dcaf17* conditional KO positive ESC clone was used as a positive control and denoted by (+) in the gel image. WT mouse genomic DNA was used for PCR as a negative control for *LoxP* sit retention and it denoted by (-) in the gel image. (E) Agarose gel image of PCR amplicons (1.95 kb) produced from the genomic DNA samples of correctly targeted F1 mice using primer set A2 and UNI to confirm correct insertion of *LoxP/FRT-Neo* cassette within the mouse genome. (F) Southern blot image confirming the genotype of positive F1 mice for correctly targeted *Dcaf17* conditional KO. Genomic DNA from positive mESC clones were used as positive control. DNA from C57Bl/6 (B6), 129/SvEv (129), and BA1 (C57Bl/6 x 129/SvEv) (Hybrid) mouse strains were used as wild type controls. The expected sizes are indicated in the image.

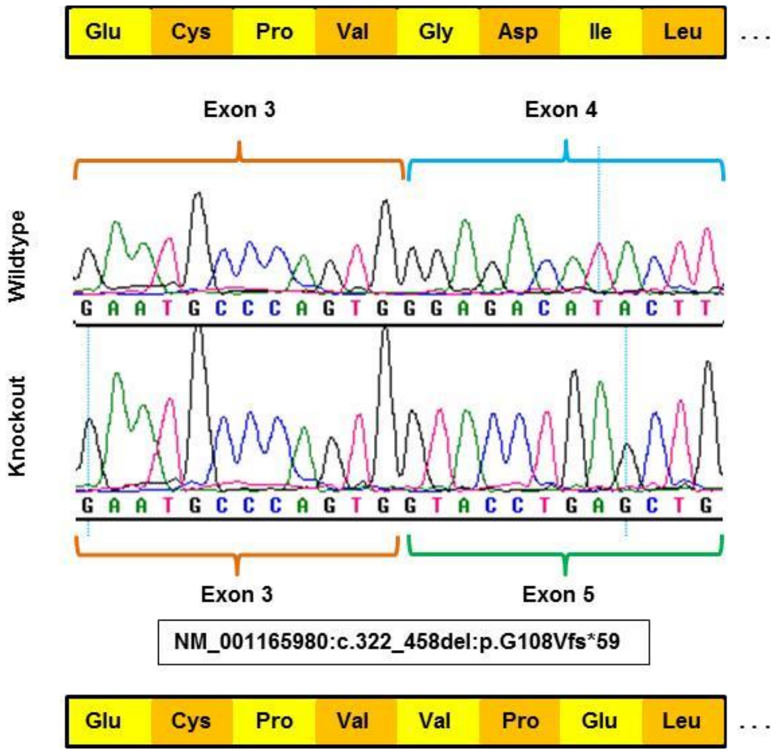

Figure S3: DNA chromatogram of reverse transcriptase (RT)-PCR sequence, performed on the *Dcaf17* transcript fragment from the *Dcaf17* KO mouse and a wild type mouse for comparison. Sequence data for the KO mouse reveals complete exclusion of exon 4 resulting in frameshift mutation (NM_001165980.1:c.322_458del:p.G108Vfs*59) with concomitant premature stop codon after 59 residues. The corresponding codons are given as alternating yellow and orange boxes.

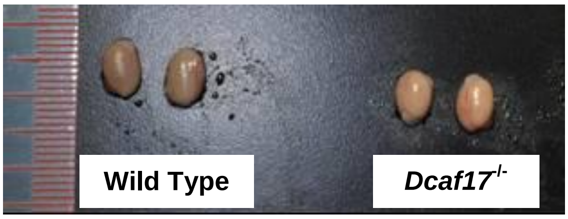

Figure S4: Morphology of testes collected from wild type and *Dcaf17*$^{-/-}$ mutant mice. No significant difference in the size and shape of the *Dcaf17*$^{-/-}$ testes were observed compare to that of wild type.

Table S1: Body weight and testis weight of wild type and *Dcaf17* KO mice.

**Phenotype**	**Genotype**		**P value (Statistical significance)**
	Wild type (*Dcaf17*$^{+/+}$)	Homozygous (*Dcaf17* $^{-/-}$)	
Average Body weight in grams ± SD	25 ± 2.45 g	26 ± 2.82 g	≤ 0.61 (Not significant)
Average Testis weight in mg ± SD	114 ± 0.0075 mg	107 ± 0.012 mg	≤ 0.37 (Not significant)

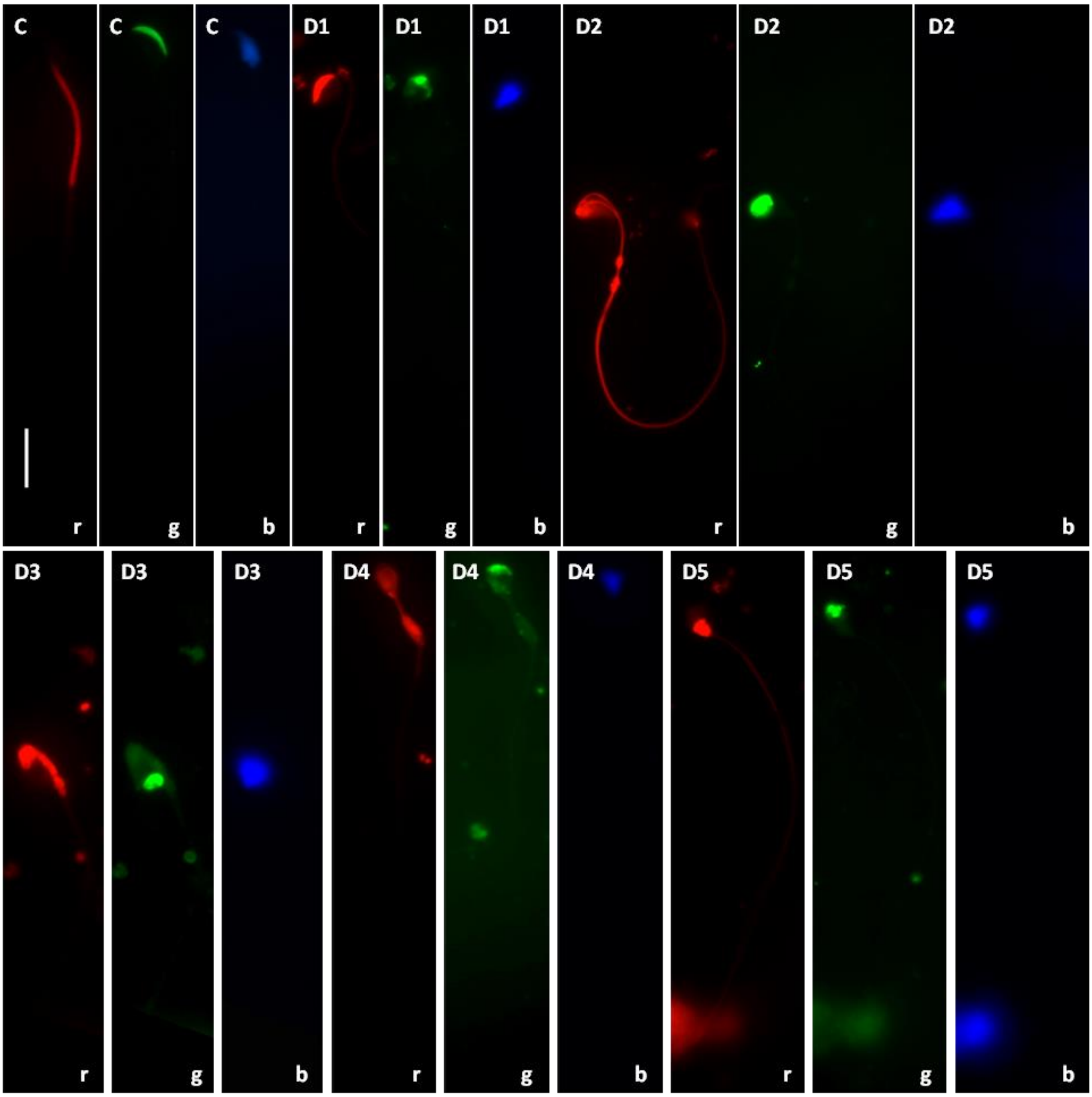

Figure S5: Fluorescence microscopy of cauda epididymal sperm from WT and *Dcaf17*$^{-/-}$ adult mice. Individual fluorescence images of fluorescently labelled wild type (C) and *Dcaf17*$^{-/-}$ (D1-5) sperm stained for acrosome (green), mitochondria (red) and nucleus (blue) corresponding to merged images shown in the figure 4 of main manuscript. Magnification is 1000X. Scale bar in the image C-r is 10 µm. r - Red; g - Green, b - Blue.

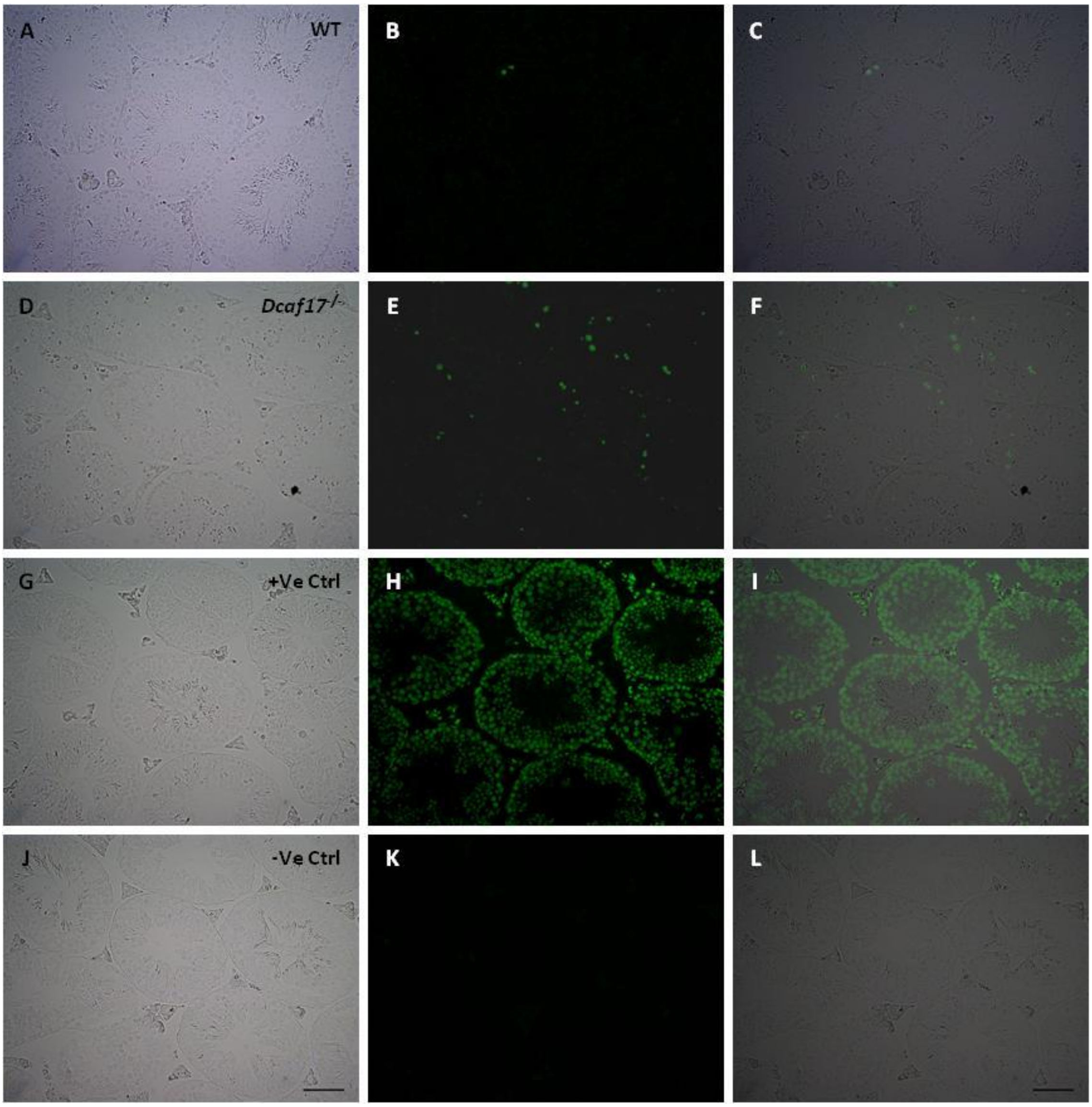

Figure S6: TUNEL staining of testes sections from 8 months old WT and *Dcaf17*$^{-/-}$ mice to assess germ cell apoptosis. Testes sections of WT (A-C) and *Dcaf17*$^{-/-}$ (D-F) mice were subjected to TUNEL assay, which detects fragmented DNA in the apoptotic germ cells (green). WT testis section (A-C) shows fewer TUNEL-positive cells (green) (B, C) compare to *Dcaf17*$^{-/-}$ testis section (E, F). Image H is positive control for TUNEL assay where the testis section was treated with DNaseI enzyme to generate fragmented genomic DNA. Image K is a negative control where testis section was treated only with labelling solution without terminal transferase. Images A, D, G and J are bright field images of respective TUNEL stained fluorescence images B, E, H, and K. Images C, F, I and L are merged images of respective bright field and fluorescence images. Magnification 200X. Scale Bar (J): 50 µm.

References

1 Southern, E. Southern blotting. *Nature protocols* **1**, 518-525, doi:10.1038/nprot.2006.73 (2006).

2 Brown, T. Southern blotting. *Current protocols in molecular biology* **Chapter 2**, Unit2 9A, doi:10.1002/0471142727.mb0209as21 (2001).
